# Size-Reduced Basis Set Calculation of Accurate Isotropic Nuclear Magnetic Shieldings Using CTOCD-GRRO and GPRO Methods in Amino Acids and Oligopeptides

**DOI:** 10.1021/acs.jpca.2c08271

**Published:** 2023-03-23

**Authors:** Michele Orza, Raphael J. F. Berger, Guglielmo Monaco, Riccardo Zanasi

**Affiliations:** † Dipartimento di Chimica e Biologia “A. Zambelli”, Università degli studi di Salerno, via Giovanni Paolo II 132, Fisciano 84084, SA, Italy; ‡ Fachbereich für Chemie und Physik der Materialien, Paris-Lodron Universität Salzburg, Jakob-Harringerstr. 2a, A-5020 Salzburg, Austria

## Abstract

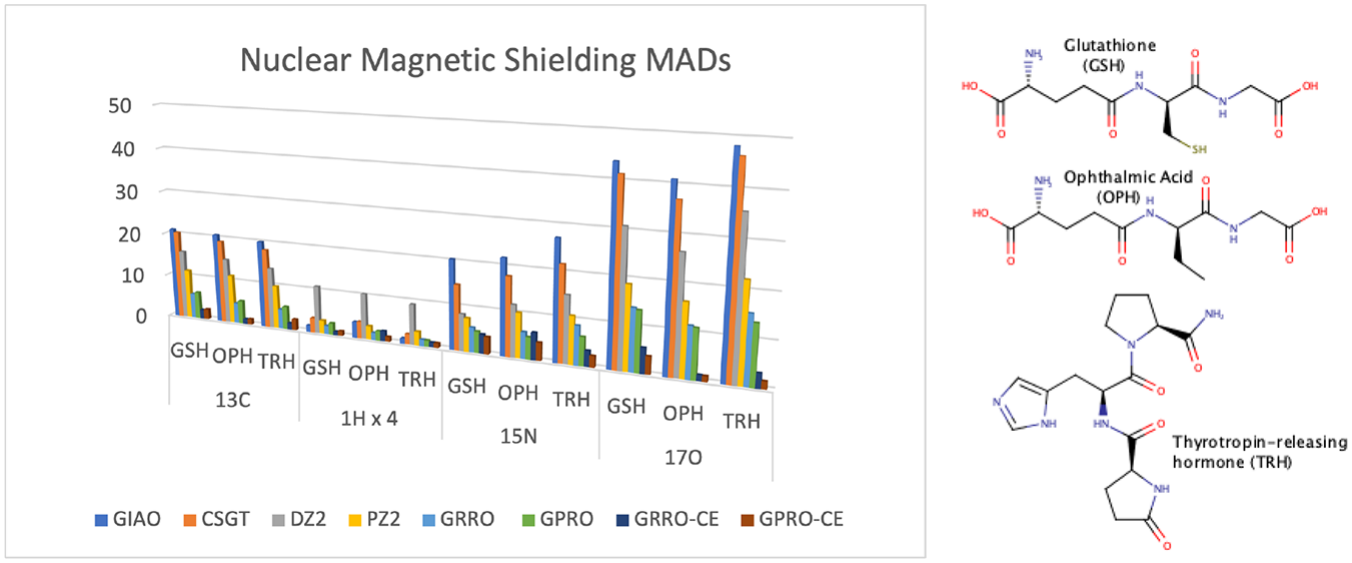

The origin-independent magnetically induced CTOCD-GRRO and -GPRO (after continuous transformation of the origin of the current density-gradient of ρ and gradient of a power of ρ) current densities are shown to vary linearly with respect to their own defining α and β parameters. The same is reflected in the connected magnetic properties, in particular the magnetic shielding. This is exploited to find values for α and β that, using small basis sets, provide isotropic nuclear magnetic shieldings matching an accurate prediction, chosen as the complete basis set limit. An application to the 20 naturally occurring amino acids shows that different nuclei require different values of the parameters, which have been determined at the BHandHLYP/6-31+G(d,p) level with or without consideration of diversified chemical environments. Using CTOCD-GRRO and -GPRO methods, equipped with the optimized parameters at this low-cost level of calculation, ^1^H, ^13^C, ^15^N, and ^17^O magnetic shielding constants in glutathione, ophthalmic acid, and thyrotropin-releasing hormone are predicted with nearly the same accuracy as that of much more expensive calculations.

## Introduction

In 1993 Keith and Bader (KB) introduced the continuous set of gauge transformations (CSGT) to define an expression for the first-order electronic origin-independent current density induced in a molecule by a static homogeneous external magnetic field, which contains explicitly an origin of the vector potential that is different for each point in space.^1^ Derivatives of the current density with respect to the inducing magnetic field components provide the current density tensor (CDT), which can be used to obtain, via integration, nuclear magnetic shielding and magnetizability tensors, avoiding the gauge-origin problem that affects the calculation of molecular magnetic properties using the so-called common origin (CO) approach.^2^

Since then, the method has received much attention, and new variants have been proposed. Our group participated in such development, contributing a family of methods known as continuous transformation of the origin of the current density (CTOCD),^3−7^ which differ by the choice of an origin function. It is convenient to recall the conventional terminology, according to which the first-order current density is given by the sum of two contributions, a paramagnetic one and a diamagnetic one, i.e., ***J***^***B***^(***r***; ***r***_0_) = ***J***_p_^***B***^(***r***; ***r***_0_) + ***J***_d_^***B***^(***r***; ***r***_0_), where ***r***_0_ is the origin of the vector potential . This notation means that both paramagnetic and diamagnetic terms are parametric functions of the vector potential origin; moreover, also the total current is shown to depend on ***r***_0_. Actually, the current density, similar to the electron density, is an origin-independent subobservable,^8^ i.e., ***J***^***B***^(***r***; ***r***_0_) = ***J***^***B***^(***r***; ***r***_0_^′^) = ***J***^***B***^(***r***), a statement that is valid only in the exact case or in the case of a calculation not affected by basis set truncation error. In practical cases adopting a finite basis set, this is no longer true and different values for the current density are calculated, shifting the origin.

In agreement with the original idea by KB,^1^ a continuous function ***d***(***r***) is used to set the origin ***r***_0_ with respect to which the current density has to be calculated. At the heart of the method there is the freedom to look for the origin function that gives point by point the best possible current density values.

So far, a number of choices for ***d***(***r***) have been proposed, with different advantages and disadvantages. For example, setting the origin in the point of evaluation itself, i.e., ***d***(***r***) = ***r***, causes both the exact and approximate diamagnetic contributions ***J***_d_^***B***^ to vanish everywhere. This choice, referred to as CTOCD-DZ1, after diamagnetic zero,^5^ also reported as *ipsocentric*,^9,10^ requires very large basis sets and it is not recommended for the calculation of accurate nuclear magnetic shieldings and magnetizabilities.^4^ To overcome this difficulty, in the original implementation of the method, an exponential function was used to shift the origin toward the nearest nucleus.^1^ This modification was termed by KB as CSDGT (continuous set of damped gauge transformations), which is also referred to in the literature as CTOCD-DZ2.^11^ Successively, the exponential function was replaced by the nuclear weight function of Becke’s algorithm^12^ for multicenter numerical integration. In this form, the method has been given the very similar acronym CSGT.^13^

Since the paramagnetic contribution ***J***_p_^***B***^ to the current density is the most difficult to calculate with high accuracy, a more suitable choice for ***d***(***r***) can be obtained by searching for an origin which causes ***J***_p_^***B***^ to vanish everywhere. The respective *Ansatz* leads to a contradiction which is resolved by setting to zero only the two components of ***J***_p_^***B***^ that are perpendicular to the inducing magnetic field.^5^ This variant of the method was termed CTOCD-PZ1, after paramagnetic zero. Similar to the previous case, a PZ2 variant exists which shifts the origin toward the nearest nucleus. These two variants of the method were found to be more accurate than the previous DZs for the calculation of magnetizability and nuclear magnetic shieldings.^6^ Recently, a few other choices for ***d***(***r***) have been developed, e.g., the CSGT with atomic size adjustments determined using the Bragg–Slater atomic radii or considering the bond critical points of the electron density distribution.^14^

In the present work we deal with the latest CTOCD schemes developed to constrain the diamagnetic induced current density vector component to be divergenceless, i.e., GRRO (after the GRadient of RhO) and GPRO (after the Gradient and a Power of RhO).^7^ Of course, the divergence of the exact total current density is equal to zero everywhere, i.e., ∇·***J***^***B***^ = 0, implying charge-current conservation. In practical cases, the divergence of the calculated current density is not zero, but it tends to vanish in the limit of a complete basis set calculation. Therefore, looking for an origin function ***d***(***r***) which minimizes, if not zeroes, the divergence of the total current density would seem a good choice leading to a faster convergence of the results. It can be shown that for any scalar differentiable function ϕ(**r**), the function ***d***(**r**) = **r** – ∇ϕ(**r**) leads to a divergenceless diamagnetic component. Rather than considering this very broad class of CTOCD methods, in ref (7), we limited the scope to look for origin functions dependent on the electron density ρ and we obtained the general form
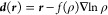
1where *f* is an arbitrary real and differentiable scalar function of the electron probability density ρ. The most simple solution is to choose *f* to be everywhere equal to a constant α, leading to the CTOCD-GRRO formulation
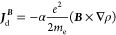
2The second scheme is obtained by a dimensional analysis, imposing *f*(ρ) = *βρ*^*n*^, where β is an adimensional constant such that *f* has the dimension of a length, i.e., [*f*(ρ) ***∇*** ln ρ] = [*L*]. This leads to the CTOCD-GPRO formulation, in which  and
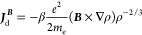
3The above ***J***_d_^***B***^ current coincides with the current density approximation obtained by Harris and Cina^15^ in the framework of a Thomas–Fermi based current density functional theory, if one sets β =  = −0.0174. Then, both CTOCD-GRRO and CTOCD-GPRO methods are defined up to a constant. Since, as we will consider in a while, α and β can take different values for different nuclei, or even the same nucleus in different chemical environments, we prefer referring to them as parameters in the following.

A first attempt to estimate α and β parameters was performed searching for the value that gives *simultaneously* the best possible calculated ^6^Li, ^15^N, ^1^H, and ^19^F magnetic shieldings (isotropic component) in the LiNHF molecule with respect to some accurate predictions. The best values turned out to be α = −0.0026 a_0_ and β = −0.0748, with the GPRO method performing better than GRRO.^7^

Already at that stage, we noted the nearly perfect linear variation of the calculated isotropic nuclear magnetic shieldings with respect to both α and β parameters, without looking for a possible explanation. Here we give a proof of such linear dependence and, as a consequence, introduce a method for the accurate calculation of isotropic nuclear magnetic shieldings at low cost. Actually, thanks to linearity, it is very easy to detect α and β values, *different* for each nucleus in a molecule, which give the exact matching with any accurate prediction, even using small basis sets and approximate methods of calculation. Obviously, upon changing the molecule, different α and β parameters for the same nucleus may result. The same can also occur changing basis set and calculation methods. We propose the following procedure to estimate isotropic nuclear shielding constants via the fitting of α and β, which will be tested and discussed in the remaining parts of this work: (i) fix a basis set of moderate size, which has been proven to provide accurate enough magnetic response properties; (ii) chose a QM method good for nuclear magnetic shielding calculations, as available within the DFT family; (iii) undertake the optimization of a minimum set of α and β parameters for some specific nuclei selected among molecules belonging to a class of systems of great interest; (iv) use these parameters to estimate the isotropic shieldings for the molecules in the class, and outside the class too, using both methods; (v) compare the results with basis set limiting values (when possible, of course) and work out mean absolute deviations (MAD); (vi) check the effectiveness of the procedure comparing MADs obtained with current standard methods for the nuclear magnetic shielding calculation, using the same QM/basis set combination. Right now, we would like to emphasize that the approach in its present form is purely theoretical. However, we rely on the very high quality of the adopted accurate predictions (see below), which is a necessary condition to have a good start. Since the same level of theory is adopted to get the accurate target predictions and to perform the fixed moderate basis set calculations, optimum α and β parameters will serve to compensate only for basis set incompleteness. For practical purposes, we plan to transform the calculated isotropic nuclear magnetic shieldings to NMR chemical shifts using linear regressions. Starting from the earlier observation by Chesnut, who demonstrated excellent correlations for isotropic shieldings with both ^1^H and ^13^C experimental chemical shifts,^16^ much work has been done to reduce not only basis set incompleteness but also systematic errors due to the many approximations retained in actual QM applications, e.g., calculation method, lacking solvent and temperature effects, by simply performing linear correlations against experimental data. These linear correlations provide very accurate results for ^1^H, ^13^C, and ^15^N.^16−24^ We are confident that incorporating such linear regressions within our approach could lead to good results. As far as it concerns the class of molecules, we thought that the set of 20 naturally occurring L-α-amino acids represents a quite convenient choice, due to the variety of nuclei and the possibility to consider further oligopeptides, as, for example, glutathione (GSH), ophthalmic acid (OPH), and thyrotropin-releasing hormone (TRH). In this context, the molecules-in-molecules fragmentation-based method applied to proteins^25^ and nucleic acids^26^ is noteworthy. In the following, we will describe the new method of calculation and the excellent results obtained, along with a way to compute the nuclear magnetic shieldings for all the nuclei of a molecule on the fly by means of Becke’s algorithm.^12^ Results will be compared with those obtained using standard and widely adopted methods, such as the already mentioned CSGT^1,13^ approach and the gauge including atomic orbital (GIAO)^13,27,28^ method.

## Theory

In the following, we assume the same notation as in ref (7). Then, for a fixed common origin ***r***_0_ the current density, induced at first-order in the electronic cloud of a molecule by an external, time-independent, homogeneous magnetic field ***B***, is given by the sum of two conventional paramagnetic and diamagnetic terms^2^
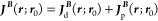
4
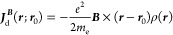
5
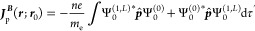
6where *n* is the number of electrons of mass *m*_e_ and charge −*e*. The first-order perturbed ground-state wave function is
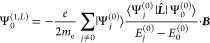
7where ***L*^** is the total angular momentum operator with respect to the common origin ***r***_0_. The ground-state probability density is
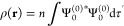
8The Born–Oppenheimer *n*-electron wave function of the molecule Ψ(**x**_1_, **x**_2_, ..., **x**_*n*_) is expressed in terms of space and spin coordinates **x**_*i*_ = **r**_*i*_ × **s**_*i*_, and integration is carried out over all electron spin coordinates and over all but one space coordinates, i.e., *dτ*′ = ds_1_dx_2_···d**x**_*n*_.

In regard to eq 4, we emphasize that the current density does not depend on the origin of the vector potential, i.e., ***J***^***B***^(***r***; ***r***_0_) = ***J***^***B***^(***r***; ***r***_0_^′^) = ***J***^***B***^(***r***). However, in a practical case, adopting numerical solution using finite basis set expansion, the common origin (CO) current density (eq 4) depends on ***r***_0_, i.e., ***J***^***B***^(***r***; ***r***_0_) ≠ ***J***^***B***^(***r***; ***r***_0_^′^).

Within the CTOCD method, a continuous shift of the origin of coordinates is assumed having the form
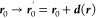
9and the current density splits into four terms
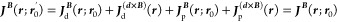
10In this way, the origin is continuously distributed and the current density turns out to be origin-independent even in approximate calculations. This nice feature was not recognized by KB in their original work^1^ nor by Lazzeretti et al. when formulating the CTOCD method.^3^ It became evident following applications, spurring for a formal proof, which was presented in ref (29).

The additional terms appearing in eq 10 with respect to eq 4 are
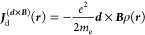
11
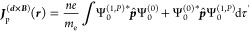
12where
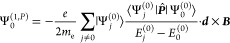
13 where ***P*^** is the total linear momentum operator.

As shown in ref (7), the ***d***(***r***) shift function for a divergenceless diamagnetic component is given by eq 1. Then, substituting eq 1 into eq 11 and summing the two diamagnetic terms in eq 10 together, one obtains (for ***r***′ = **0**)
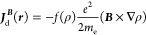
14which corresponds to both eq 2 and eq 3 for the two forms *f* = α and , which we refer to as GRRO and GPRO, respectively.

Substituting eq 1 into eq 13, we can now see how the paramagnetic term transforms. The first-order perturbed wave function Ψ_0_^(1,*P*)^ splits in two pieces
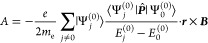
15
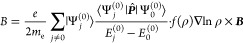
16which provide two ***J***_p_^(***d*** × ***B***)^ terms
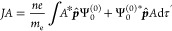
17
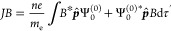
18The former is exactly the current term that replaces the conventional diamagnetic current in the DZ1 method. The additional contribution *JB* is clearly proportional to the parameters α and β for the GRRO and GPRO method, respectively. Therefore, as it can be easily seen, when α and β are set to zero both GRRO and GPRO currents reduce to DZ1, as required by putting *f* = 0 in eq 1. To have GRRO and GPRO operative, a value must be assigned to α and β, which must both be lower than zero to keep the tropicity of the diamagnetic vortex close to any nucleus. When such negative values are assigned to the parameters, a contribution proportional to either α or β is added to the DZ1 current via eqs 14 and 18.

The same happens to any magnetic property that can be obtained from the current density. In particular, any magnetic shielding tensor element can be calculated via the Biot–Savart law,^30,31^ integrating the appropriate current density tensor element multiplied by a geometrical factor
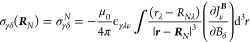
19where ***R***_*N*_ is the position of nucleus *N*, ϵ_*γλν*_ is the Levi–Civita tensor, and Einstein implicit sum over repeated Greek indices is assumed. Chemical shifts are related to the isotropic component
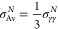
20

Therefore, replacing GRRO or GPRO currents in eq 19, a nuclear magnetic shielding constant can be calculated via eq 20, which varies linearly with respect to α or β. As a consequence, there is the concrete possibility to optimize α and β for any nucleus in a molecule, making the isotropic component coincident with any accurate prediction or experimental derived magnetic shielding. Considering eq 19, one may ask whether the proposed method can be used to calculate the full shielding tensor, for example for simulating solid-state NMR or analyzing the shielding tensor anisotropy. Very preliminary tests have shown that our approach cannot be used profitably for this purpose. On the other hand, one single parameter, α or β, can fix only one number. To get the full tensor, a more sophisticated function *f*(ρ) in eq 1, containing more degrees of freedom, should be devised.

## Methods

The chemical elements present in every amino acid are C, H, N, and O, with the addition of S in cysteine and methionine. The isotopes ^1^H and ^13^C are the most frequently used in NMR spectroscopy, also ^15^N is commonly used, whereas ^17^O finds very special applications in biochemical studies as well as ^33^S, due to their 5/2 and 3/2 spins and low abundance. However, for the purposes of this paper, we have optimized α and β parameters for all these five elements. The procedure we adopted to determine a minimum set of values, good for all amino acids, is described below.

In order to fix a basis set of moderate size for the calculation protocol, we relied on the outstanding work by the Tantillo group.^22,24^ Following their recommendation, we have considered the 6-31+G(d,p) basis set^32−38^ to be adequate for our purposes. This is crucial for keeping the calculation efforts as small as possible. As it concerns the choice of the density functional to perform the QM calculations for good magnetic response, we relied on our previous experience,^39^ according to which the BHandHLYP^40^ is appropriate for the calculation protocol, emphasizing that the central quantity here is the current density, from which the isotropic nuclear magnetic shieldings can be obtained by integration. Moreover, as shown by Lehtola et al.,^41^ the BHandHLYP functional provide the most accurate magnetizability over a set of 51 density functional approximations.

For the accurate prediction to compare with the results, searching for a basis set capable of providing isotropic shielding estimates equivalent to the complete basis set limiting values would be ideal. The condition to establish such an equivalence is provided by the fact that all GIAO, CSGT, and CTOCD methods provide the same results for the complete basis set at the same level of QM calculation. Therefore, what we did was to look for a basis set large enough, but feasible for all the 20 amino acids, which gives the same isotropic nuclear magnetic shieldings for all GIAO, CSGT, and CTOCD methods within a desired accuracy and to take these results as good complete basis set estimates. Among the many possibilities, we found that the pcSseg-4 basis set^42^ gives this matching of GIAO, CSGT, and CTOCD estimates up to two decimal digits for all nuclei of all amino acids. Complete basis set limits are collected under the heading λ in all tables reported within the Supporting Information. To evaluate the performance of the proposed method, mean absolute deviations (MAD) from λ are obtained and compared with those affecting standard nuclear magnetic shielding calculation methods, such as GIAO^13,27,28^ and CSGT.^1,13^ Defining the absolute deviation (AD)
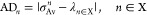
21for a particular nucleus *n* from the set X = {^13^C, ^1^H, ^15^N, ^17^O, ^33^S}, MADs are calculated averaging AD_*n*_ over all nuclei of the same type in each molecule.

Gaussian 16^43^ has been used to calculate GIAO and CSGT nuclear magnetic shieldings, as well as the perturbed wave functions for the subsequent CTOCD calculations, which were performed using SYSMOIC.^44,45^ As for the molecular geometries, we opted for the simpler solution, i.e., for each amino acid we took the most stable neutral conformation using the Spartan 02 program at the MMFF94 force field level of theory.^46−50^

According to the linear dependence proven in the previous section of GRRO and GPRO current densities on their own defining parameters, for each atom in all 20 amino acids, α and β values that make 6-31+G(d,p) σ_Av_^*n*^ perfectly equal to the accurate predictions, obtained using the pcSseg-4 basis set, have been determined using a two-point analytical formula (see Figure S1 in the Supporting Information for a couple of examples). In this way we obtained a rather large set of nonaggregated data ready to be analyzed statistically and/or on the basis of chemical similarity. This data set of α and β values, which we refer to as a primitive data set, is available upon request. Of course, the convenience of the method is based on the possibility of reducing the number of parameters to a minimum number, keeping great accuracy even changing the molecular conformations. This latter point will be tested performing calculations on some oligopeptides, in which the conformation of the constituent amino acids is largely different from that considered during the optimization process, and on nine additional conformations of alanine obtained rotating independently the −CH_3_, −COOH, and −NH_2_ groups about the single bonds connecting the fragments to the central α-carbon by 40°, 80°, and 120°.

Furthermore, we have tried to achieve the even more challenging goal of performing the calculation using only one integration step for all nuclei at once, by means of a specific implementation of Becke’s algorithm.^12^ As is very well-known, the algorithm deals with the evaluation of three-dimensional integrals of the type
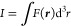
22and assumes that a *relative weight* function *w*_A_(***r***) can be assigned to each atom A in the molecule such that
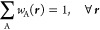
23and such that each *w*_A_(***r***) is equal to unity in the vicinity of its own nucleus and vanishes in a continuous and well-behaved manner near any other nucleus. The molecular function *F*(***r***) is decomposed into single-center components
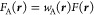
24since, as it can be easily proven
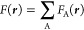
25Therefore, eq 22 reduces to a sum of *single*-center integrations *I*_A_ over each atom in the molecule:
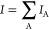
26where
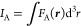
27In our specific case, for the calculation of the magnetic shielding constant (eq 20), we have that
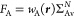
28where
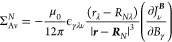
29is the isotropic component of the magnetic shielding density function.^30,31^ The key step is to use the same grid of current density tensor elements  for all the nuclei in the molecule by means of eq 29 and carry on the integration in parallel. However, calculating the GRRO or GPRO current density setting the α or β parameter to a value optimized for one specific nucleus, let us say *M*, will lead to only one accurate magnetic shielding, i.e., σ_Av_^*M*^. Nonetheless, thanks to function *w*_A_ that gives a substantial weight only to points within the boundary of its own nucleus A, we observe that when *N* ≠ A the magnitude of Σ_Av_^*N*^ at those points is greatly reduced by a factor . Therefore, instead of using only a single α/β value for the calculation of the GRRO/GPRO current density grid, the parameter is set differently for each single-center integration *I*_A_, putting it equal to the value optimized for atom A. This multiple setting allows calculating at once in a single integration run all nuclear magnetic shieldings, which are affected just by a small deviation from their optimum value. One further advantage of this procedure is that more values for α and β can be included within the data table for the same nucleus, which might take care of different substitution patterns and even of different kinds of atomic hybridization. This last possibility would ensure greater precision, compensating for the loss of accuracy due to the use of multiple parameter values during the integration procedure.

## Results and Discussion

The first attempt we made was to reduce the number of parameters in the primitive data set, calculating one average value for each chemical element, without considering any distinction for different substitutions. The α and β values obtained by averaging the 107 carbons, 197 hydrogens, 29 nitrogens, 49 oxygens, and 2 sulfurs are reported in Table 1, together with respective standard deviations. It can be observed that (i) α̅ values for protons are 2 orders of magnitude larger than those for heavier nuclei; (ii) β̅ values are more flat and stay in a range encompassing the single value optimized previously;^7^ (iii) standard deviations for heavier nuclei are relatively small in comparison with mean values; (iv) for protons, standard deviations are much higher, revealing a fairly large range of optimized values. In addition, we observed that the linear dependence slopes depend on the chemical element, but not so much on the chemical environment. For example, the GRRO  and GPRO  slopes are found to be nearly −23750 *a*_0_^–1^ ppm and −2400 ppm, respectively, for all carbon atoms, while they are nearly −11 *a*_0_^–1^ ppm and −60 ppm for hydrogen atoms. This means that despite the large standard deviations determined for hydrogen atoms, the error on the σ_Av_^H^ estimates is predicted to be only 0.6 ppm for both methods (11 × 0.05, 60 × 0.011). On the other hand, the error for σ_Av_^C^ estimates is ∼3.8 ppm for GRRO and GPRO (23750 × 0.00016, 2400 × 0.0016). For N and O, slopes are higher especially for the GRRO method in the case of O, leading to larger errors. For S, the data are too few. These error estimates can be readily confirmed running one separate integration step for each nucleus using the corresponding α̅ or β̅ value reported in Table 1. We stress that α and β values depend on basis set choice and the QM method of calculation. Therefore, their physical meaning, restricted to that basis set, is that they compensate for the error introduced by the truncated basis set. Furthermore, as it must be, for each QM method improving the basis set leads to a lower slope of the linear dependence of the GRRO and GPRO current densities on their respective parameters, which can have any value within the basis set limit.

**Table 1:** BHandHLYP/6-31+G(d,p) Mean Values of GRRO and GPRO Parameters for the Chemical Elements in the 20 Natural Amino Acids

nucleus	α̅ /*a*_0_	β̅	number of
^13^C	–0.00680 ± 0.00016	–0.0674 ± 0.0016	107
^1^H	–0.37 ± 0.05	–0.061 ± 0.011	197
^15^N	–0.00545 ± 0.00016	–0.074 ± 0.002	29
^17^O	–0.0041 ± 0.0002	–0.073 ± 0.004	49
^33^S	–0.00170 ± 0.00003	–0.1117 ± 0.0017	2

A second attempt we made was to consider the different chemical environment of each nucleus. Actually, inspecting the data set of primitive α and β parameters, several significant differences can be observed. For example, the α and β values for carboxylic protons turn out to be −0.274 ± 0.007*a*_0_ and −0.037 ± 0.002, respectively, while their mean value for H_α_’s is −0.328 ± 0.016*a*_0_ and −0.061 ± 0.005 (here H_α_ indicates the hydrogen attached to the asymmetric carbon). Many other examples can be found that we do not report here for the sake of space (available upon request). These rather large differences account for the large standard deviations observed in Table 1, which may be reduced significantly by differentiating the nuclei for the chemical environment. For this reason, we wrote a simple algorithm that goes through the tree of molecular bonds checking the substituents of each atom and grouping them in agreement with standard terminology. The chemical environment of heavy atoms has been identified by nearest-neighbors, in substantial analogy with Benson’s group additivity rules for thermodynamics.^51^ For hydrogens, in order to have transferable values of the parameters, first and second non-nearest neighbors had to be used, which finds some analogy in empirical methods to predict chemical shift in proteins.^52^ The α and β parameters determined for different chemical environments are given in the Supporting Information. In its present form, the algorithm is able to distinguish up to 30 different kinds of protons, 26 carbon types, 12 nitrogen, and 9 oxygen types, which is more than what we need for the amino acids here considered. For each recognized type of atom, different α and β values are assigned, recovered from the primitive data set as mean value for the corresponding chemical environment. In the following they will be labeled as α̅_CE_ and β̅_CE_ for “chemical environment”.

Nuclear magnetic shieldings have been calculated for all amino acids using eight different methods, i.e., GIAO, CSGT, DZ2, PZ2, GRRO, and GPRO, the latter two adopting the α̅ and β̅ parameters reported in Table 1 and the α̅_CE_ and β̅_CE_ obtained by differentiating for chemical environment. The full set of results is given within the Supporting Information, together with the pcSseg-4 results, which appear under the heading λ. Here we report and comment on MADs calculated by averaging the absolute deviations (eq 21).

**Figure 1:**
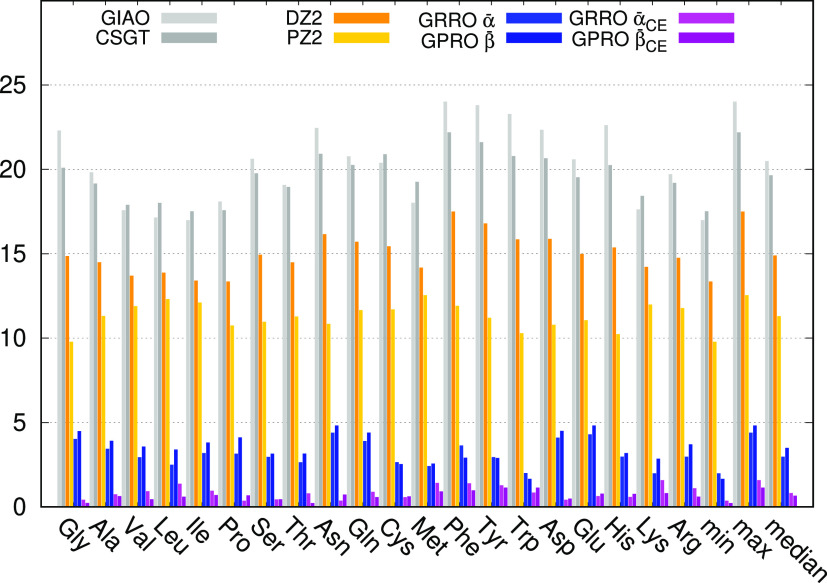
BHandHLYP/6-31+G(d,p) mean absolute deviation in ppm for ^13^C.

Let us start with MADs obtained for ^13^C illustrated in Figure 1 (the full set of data is reported in Table S26 of the Supporting Information). Owing to the small basis set size, somewhat large MADs, with a median of nearly 20 ppm, can be observed for both GIAO and CSGT methods, which perform equivalently. Slightly better is the performance of DZ2, while PZ2 almost halves the GIAO MADs. Remarkably, the very simple GRRO and GPRO approaches utilizing the few parameters reported in Table 1, i.e., only one parameter for each nucleus type, provide an amazing performance, which touches the predicted accuracy. In this case GRRO is slightly better than GPRO. These already good results are further improved by differentiating the parameters for chemical environment (see light and dark pink bars corresponding to the last two columns of Table S26). In this case, GPRO performs better that GRRO, showing a median which comes very close to 0.5 ppm.

**Figure 2:**
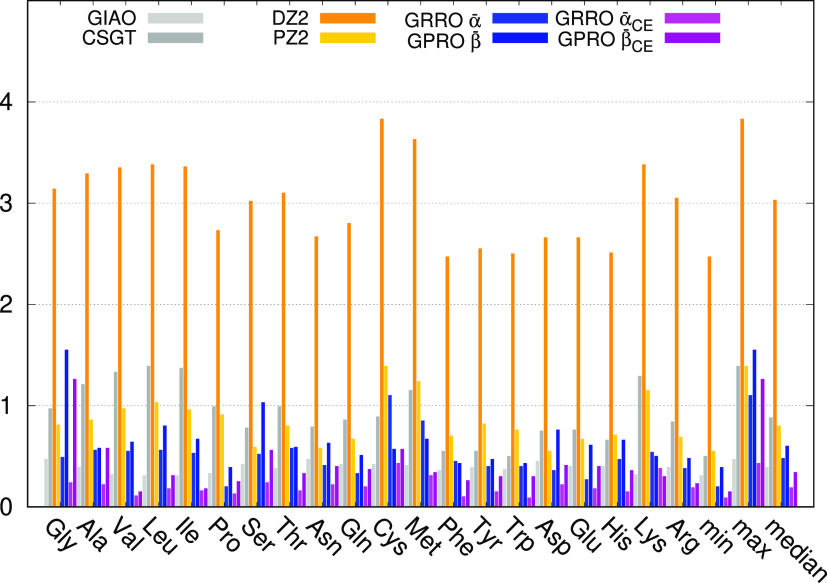
BHandHLYP/6-31+G(d,p) mean absolute deviation in ppm for ^1^H.

MADs calculated for the proton magnetic shielding constants are illustrated in Figure 2 (corresponding data are reported in Table S27). In this case, the GIAO method performs much better than in the previous case and proves superior to CSGT, DZ2, and PZ2, a result often underlined in the literature (see for example ref (13)). However, the simpler GRRO and GPRO show again an excellent performance, getting very close to the GIAO accuracy, which is surpassed by both the GRRO and GPRO when using chemical environment differentiation. The MAD median of only 0.19 ppm for the GRRO associated with α̅_CE_ underlines the superiority of this approximation.

**Figure 3:**
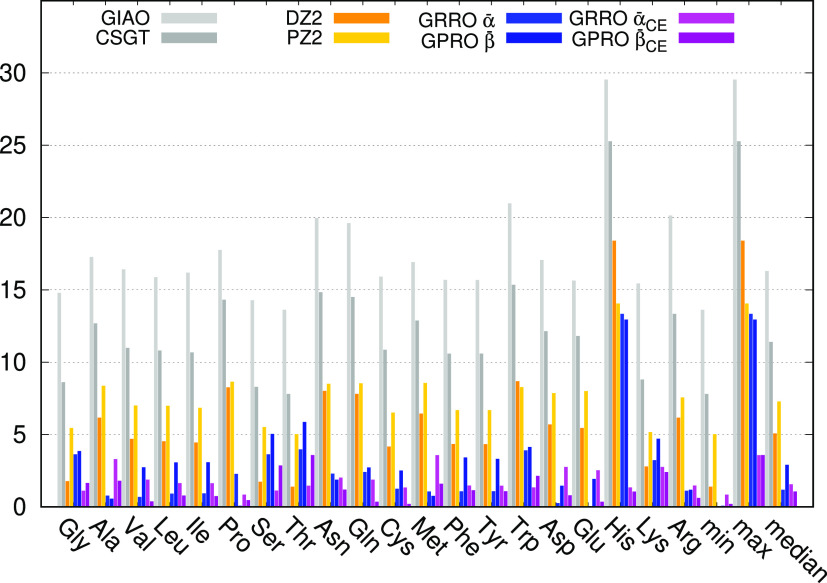
BHandHLYP/6-31+G(d,p) mean absolute deviation in ppm for ^15^N.

For ^15^N (see Figure 3 and Table S28), MADs resemble those of carbon with the GIAO showing the larger deviations. In this case, it is worth noting that histidine (His) gives the maximum deviations for all methods but GRRO and GPRO with chemical environment differentiation. This is evident for all and in particular for the simpler GRRO and GPRO methods using α̅ and β̅, for which the median is still quite small. Looking within the data set of primitive parameters, we observe that the nonprotonated nitrogen of the imidazole side chain of histidine displays both α and β parameters which are quite different from those of the other nitrogen atoms. Then, owing to the larger number in total of non-aromatic nitrogen atoms, α̅ and β̅ mean values turn out to be very different from the α and β of the imidazole nonprotonated nitrogen. Consequently the calculated magnetic shielding turns out to be quite poor. Interestingly, this kind of nitrogen atom is problematic also for GIAO, CSGT, DZ2, and PZ2 methods. Remarkably, when the chemical environment is taken into account, deviations for histidine reduce to nearly 1 ppm.

**Figure 4:**
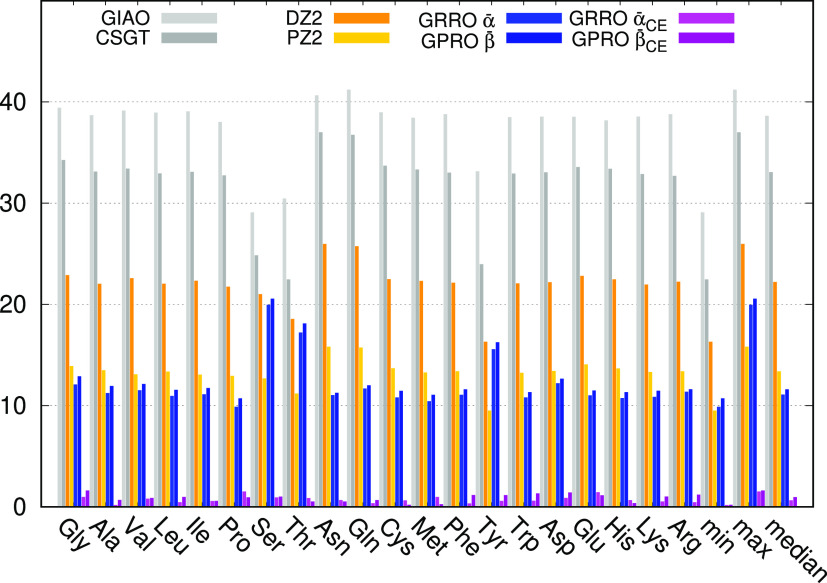
BHandHLYP/6-31+G(d,p) mean absolute deviation in ppm for ^17^O.

For ^17^O (see Figure 4 and Table S29), the discussion is similar to the previous ones concerning GIAO, CSGT, DZ2, and PZ2, with GIAO the worst, CSGT a little better, PZ2 definitively better than both, and DZ2 somewhere in between. Looking at GRRO and GPRO using α̅ and β̅, it can be seen that MADs are almost one order of magnitude larger than those observed before. The reason is a simple one. For the neutral, e.g., not zwitterionic form, amino acids here considered, there are mainly two kinds of oxygen, one carbonylic and one hydroxylic in the same carboxylic group. They have different α and β parameters, and using α̅ and β̅ mean values in GRRO and GPRO methods, respectively, does not provide a good result for both of them. As clearly recognizable looking at the light and dark pink bars and data reported in the last two columns of Table S29, chemical environment differentiation gives very good results, with a median which is less that 1 ppm for both GRRO and GPRO. F-tests indicate that the better agreements obtained by GRRO and GPRO methods either with or without adjustment for the chemical environment are statistically significant at the 99% level (see Tables S37 and S38).

In actual applications, calculation of relative chemical shifts must be carried out. As already mentioned previously, the present approach can only compensate for basis set incompleteness. Then, a suitable transformation of isotropic shieldings to relative chemical shifts, able to reduce a number of other systematic errors, can be obtained by means of linear correlations against experimental data.^16−24^ Of course, such linear correlations are routinely applied also to GIAO and CSGT predictions. Therefore, a question arises: does the greater accuracy of GRRO and GPRO estimates of isotropic ^13^C, ^15^N, and ^17^O with respect to GIAO and CSGT predictions for the amino acids discussed above still persist? We attempted to answer by performing a direct transformation of the calculated σ_Av_^*n*^ in relative chemical shifts δ_*n*_ by using the formula^22^ δ_*n*_ = σ_Av,ref_^*n*^ – σ_Av_^*n*^. Since specific errors in computed isotropic shielding constants for the carbon atoms attached to silicon in TMS can adversely affect chemical shifts computed in this manner,^22^ we have opted for a different reference compound such as propane and focused on ^13^C and ^1^H. In this way, neither GIAO nor GRRO/GPRO are privileged. The σ_Av,ref_^*n*^ has been calculated for both large and small basis sets; then MADs for isotropic shieldings collected in Tables S26 and S27 have been converted in MADs for relative chemical shifts by means of a straightforward manipulation. These MADs are reported in the Supporting Information within Table S39 for ^13^C and Table S40 for ^1^H. As can be observed, GIAO gains greatly in accuracy for both nuclei, while GRRO and GPRO lose in accuracy using α̅ and β̅, a little less using chemical environment differentiation. All that can be conveniently summarized considering the medians only, which are given here in Table 2. For ^13^C the GIAO median MAD reduces by almost three times passing from isotropic shieldings to relative chemical shifts. However, although GRRO-α̅ and GPRO-β̅ median MAD almost double, they remain lower than GIAO by at least 2 ppm. GRRO and GPRO with chemical environment differentiation remain quite accurate also in terms of relative chemical shifts. For ^1^H the GIAO median MAD halves passing from isotropic shieldings to relative chemical shifts, while GRRO and GPRO get a little worse in all variants.

**Table 2:** MAD Medians in ppm for Relative Chemical Shift and Isotropic Shielding

		GRRO	GPRO	GRRO	GPRO
	GIAO	α̅	β̅	α̅_CE_	β̅_CE_
δ_^13^C_	7.7	5.9	4.8	0.7	1.5
σ_Av_^^13^C^	20.5	2.9	3.5	0.8	0.6
δ_^1^H_	0.2	1.2	0.6	0.6	1.3
σ_Av_^^1^H^	0.4	0.5	0.6	0.2	0.3

To verify the usefulness of the optimization work shown above for the single amino acids, we have used the calculation procedures for determining ^13^C, ^1^H, ^15^N, and ^17^O isotropic magnetic shielding constants in three oligopeptides: glutathione (GSH), ophthalmic acid (OPH), and thyrotropin-releasing hormone (TRH). Since the conformation of the amino acids contained in the oligopeptides is not the same as in the individual cases, the conformation dependence of α̅ and β̅, with and without chemical environment differentiation, has been tested. As before, basis set limit values have been obtained adopting the pcSseg-4 basis set. Larger oligopeptides would have required much greater computer resources. Notwithstanding, the choice seemed quite good to us.

Calculated nuclear magnetic shielding constants for GSH, OPH, and TRH are given within the Supporting Information. MADs relative to the eight calculation methods are reported in Table 3. As can be observed, reported MADs parallel those of the single amino acids, e.g., GIAO is superior to CSGT, DZ2, and PZ2 only in the proton case, while it is always the worst for the other nuclei. GRRO and GPRO, with or without chemical environment differentiation, are only slightly larger that those of the individual amino acids, revealing an encouraging insensitivity to conformation. In general, both GRRO and GPRO approaches appear to be equivalent and provide good accuracy, except ^17^O, already using the α̅ and β̅ mean values reported in Table 1. Introducing chemical environment differentiation, median deviations from the basis set limit reduce to only ∼1 ppm for ^13^C, 0.27 ppm for ^1^H, ∼4 ppm for ^15^N, and ∼2.5 ppm for ^17^O.

**Table 3:** BHandHLYP/6-31+G(d,p) Mean Absolute Deviation in ppm for Glutathione (GSH), Ophthalmic Acid (OPH), and Thyrotropin-Releasing Hormone (TRH)

						GRRO	GPRO	GRRO	GPRO
nuc	OP	GIAO	CSGT	DZ2	PZ2	α̅	β̅	α̅_CE_	β̅_CE_
^13^C	GSH	21.00	20.34	15.81	11.32	5.76	6.17	2.03	2.30
	OPH	20.66	19.17	14.96	11.20	4.69	5.34	1.13	1.24
	TRH	20.02	18.23	13.96	9.90	4.51	5.24	1.48	2.52
^1^H	GSH	0.38	0.84	2.73	0.76	0.48	0.66	0.20	0.27
	OPH	0.92	0.96	2.60	0.77	0.41	0.55	0.59	0.28
	TRH	0.31	0.59	2.32	0.79	0.37	0.35	0.27	0.26
^15^N	GSH	20.40	14.88	8.41	7.60	5.63	4.86	4.33	3.90
	OPH	21.86	17.93	11.78	10.16	5.98	5.00	6.14	4.05
	TRH	27.20	21.71	15.20	10.80	8.82	6.48	3.59	2.58
^17^O	GSH	43.78	41.30	30.82	18.85	13.98	13.59	5.70	3.98
	OPH	40.96	37.03	26.55	16.40	11.53	11.20	1.30	1.23
	TRH	48.17	46.31	35.61	22.18	15.40	13.58	3.27	1.72

Computer efforts are immeasurably lower. For example, the ratio of the elapsed times for the two basis sets pcSseg-4 and 6-31+G(d,p), at the same conditions, e.g., same machine, same number of processors, same functional etc., is >1000 on average, which obviously is simply some power of the ratio of the number of basis functions in the two types of calculations.

To examine further the conformation dependence of α and β parameters, we have considered how MADs change rotating the −CH_3_, −COOH, and −NH_2_ groups in alanine. Three torsional angles differing by 40°, 80°, and 120° have been chosen and applied to each group independently of each other, leading to nine additional conformations, which represents already a considerable amplification of data. Large pcSseg-4 and small 6-31+G(d,p) basis set calculations have been performed to get λ limiting results and GIAO, CSGT, GRRO, and GPRO predictions, from which MADs have been calculated and reported in the Supporting Information within Tables S41–S44 and relative plots for ^13^C, ^1^H, ^15^N, and ^17^O, respectively. In all examined cases MADs are fairly constant, confirming a substantial insensitivity to conformation.

### How Is the Performance of the Method in Non-amino Acid Molecules?

Considering the set of the 20 natural occurring amino acids seemed to us a pretty good choice, especially because it opens the way toward the application of the proposed method to highly interesting polypeptide macromolecules, for which the computational effort would be too high using currently available calculation approaches for accurate nuclear magnetic shielding determination. However, it seemed useful to evaluate the performance of the method also in the case of molecules other than amino acids to verify possible applicability limits. To this purpose, we have taken into account a selection of molecules common to the NS372 benchmark set reported in ref (53) and the reference benchmark set in ref (54), which contain ^13^C, ^1^H, ^15^N, and ^17^O nuclei. These are the 12 molecules: C_2_H_4_, C_2_H_4_O, C_3_H_4_, CH_4_, CO, H_2_CCO, H_2_CO, H_2_O, HCN, N_2_, N_2_O, and NH_3_. For them, calculated isotropic shieldings are collected in Table S36 of the Supporting Information. For each atom the accurate prediction, determined using a BHandHLYP/pcSseg-4 combination of functional and basis set, is compared with GIAO, GRRO, and GPRO predictions calculated, as before, at the BHandHLYP/6-31+G(d,p) level. The latter two have been obtained using α̅ and β̅ reported in Table 1, i.e., without chemical environment differentiation. From the ADs reported in the last three columns of Table S36, MADs have been evaluated and reported in Table 4. As can be observed, ^13^C GRRO and GPRO MADs indicate a good behavior, being three times lower than GIAO and only a little higher than those reported in Table 3 for oligopeptides. Conversely, for ^1^H, GRRO and GPRO MADs are much worse than GIAO and those obtained for oligopeptides too, revealing that proton α̅ and β̅ parameters require different work of optimization, using a different primitive data set at least. For ^15^N, although GRRO and GPRO MADs are better than GIAO, the comparison with the deviations obtained for amino acids and oligopeptides indicates the same conclusion that a different optimization of the parameters must be carried out. This is quite evident considering also the large GRRO and GPRO MADs obtained for ^17^O. In summary, although the parameters of the GRRO and GPRO methods should be optimized for chemically similar systems, the α̅ and β̅ values optimized here for amino acids already provide nuclear shieldings that, compared with GIAO, indicate a sizable improvement for ^13^C, some improvement for ^15^N, a modest worsening for ^17^O, and a sizable worsening for ^1^H.

**Table 4:** BHandHLYP/6-31+G(d,p) Mean Absolute Deviation in ppm for the Selected Non-amino Acid Molecules

nuc	GIAO	GRRO	GPRO
^13^C	22.66	6.84	6.81
^1^H	0.30	1.17	2.56
^15^N	31.33	19.48	20.70
^17^O	27.96	35.49	33.87

## Conclusions

The linear dependence of the GRRO and GPRO current density (and, therefore, of nuclear magnetic shielding constants) on their own defining α and β parameters^7^ has now been proven. It is shown how this linear dependence can be exploited to introduce a new method for the inexpensive calculation of accurate isotropic nuclear magnetic shieldings using a small basis set at the DFT level of approximation. The method is based on the determination of a primitive data set of parameters (which depends on the adopted basis set and functional) that allows reproducing any accurate prediction.

An application of the method is reported for the 20 naturally occurring L-α amino acids, determining a set of GRRO and GPRO α̅ and β̅ average values adopting the BHandHLYP/6-31+G(d,p) combination of density functional and basis set. Using these α̅ and β̅ parameters, with and without chemical environment differentiation, the isotropic shieldings of the 20 amino acids show mean absolute deviations with respect to the accurate predictions, represented by the limit values obtained using the pcSseg-4 basis set, of only few ppm for heavier nuclei and as small as 0.19 ppm for protons. The new procedure is further tested by calculating the isotropic shieldings of glutathione (GSH), ophthalmic acid (OPH), and thyrotropin-releasing hormone (TRH), keeping the high accuracy obtained for the single amino acids; only a very weak increment of the deviations with respect to the accurate prediction is observed, which is unlikely to be due to conformation effects since the latter have been found to be negligible. For the amino acids and oligopeptides studied in the present work, the new procedure provides isotropic nuclear magnetic shieldings that are much more accurate than those calculated adopting standard calculation methods, such as GIAO and CSGT, using the same functional and 6-31+G(d,p) basis set.

Changing basis set and or functional, a new set of α̅ and β̅ parameters, with and without chemical environment differentiation, has to be determined, but this does not seem to be a major conceptual problem. However, trying to use a single small set of GRRO and GPRO parameters for the same nuclei in different classes of molecules is proven to provide outcomes that are significantly improved over GIAO only for ^13^C. For other nuclei, the method should be addressed differently, for example, differentiating for chemical environment. We would like to stress that the proposed approach reduces only basis set incompleteness. Linear correlations of GRRO/GPRO isotropic shieldings against experimental chemical shifts should be applied to reduce other systematic errors.

Eventually, we would like to underline that the poor performance of the GIAO approach for C, N, and O reported here is mainly due to the inadequacy of the 6-31+G(d,p) basis set, which is not even optimized for magnetic shielding calculations. Usually, GIAO performs much better.^13^

Last but not least, the procedure described in this work is readily at hand to all Gaussian^43^ users by means of a couple of simple commands, as described in ref (45). Then, nuclear magnetic shieldings can be calculated for all nuclei of a molecule on the fly, thanks to a modified Becke’s algorithm of integration.

## Data Availability

All relevant data and programs can be downloaded from our server via links: http://sysmoic.chem.unisa.it/WORKED_EXAMPLES/Supp-Mat/ for data and scripts and http://sysmoic.chem.unisa.it/DISTRIB/ for the freely available SYSMOIC programs package.^44,45^ A zipped file can be downloaded from the first link with a hierarchy of folders for the 20 amino acids. Each folder contains: (i) input data to run BHandHLYP/6-31+G(d,p) calculations of nuclear magnetic shieldings and perturbed MO coefficients using the Gaussian 16 suite of programs;^43^ (ii) a shell script to calculate nuclear magnetic shieldings using the GRRO and GPRO methods, as newly implemented within the SYSMOIC package.^44,45^ Instructions on how to run the scripts can be found within the README.txt file in the top folder. Output files containing the results reported in the Supporting Information are also provided, which can be easily reproduced running the shell scripts. From the second link the SYSMOIC package can be anonymously accessed and installed on three different platforms (Linux, Mac, and Windows systems). Of course, the installation is mandatory to run the scripts that perform GRRO and GPRO calculations. A manual for installing and using SYSMOIC can be found online (see ref (44)).

## Supporting Information Available

Cartesian coordinates and calculated isotropic nuclear magnetic shielding constants (all methods and basis sets) for all molecules taken into account; tables giving (i) mean absolute deviations of σ_Av_ from accurate predictions; (ii) GRRO and GPRO α̅_CE_ and β̅_CE_ for ^13^C, ^1^H, ^15^N, ^17^O, and ^33^S; and (iii) sum of squares of the residuals for F-tests (PDF)
