# Supplementary material for: Size-Reduced Basis Set Calculation of Accurate Isotropic Nuclear Magnetic Shieldings Using CTOCD-GRRO and GPRO Methods in Amino Acids and Oligopeptides
Source: J Phys Chem A. 2023 Mar 23;127(13):3036–47. doi: 10.1021/acs.jpca.2c08271 (PMC10084456; doi:10.1021/acs.jpca.2c08271)

Supporting Information

Size-Reduced Basis Set Calculation of Accurate Isotropic Nuclear Magnetic Shieldings Using CTOCD-GRRO and GPRO Methods in Amino Acids and Oligopeptides

Michele Orza,† Raphael Berger,‡ Guglielmo Monaco,† Riccardo Zanasi†

† Dipartimento di Chimica e Biologia "A. Zambelli", Università degli studi di Salerno, via Giovanni Paolo II 132, Fisciano 84084, SA, Italy

‡ Fachbereich für Chemie und Physik der Materialien, Paris-Lodron Universität Salzburg, Jakob-Harringerstr. 2a, A-5020 Salzburg, Austria

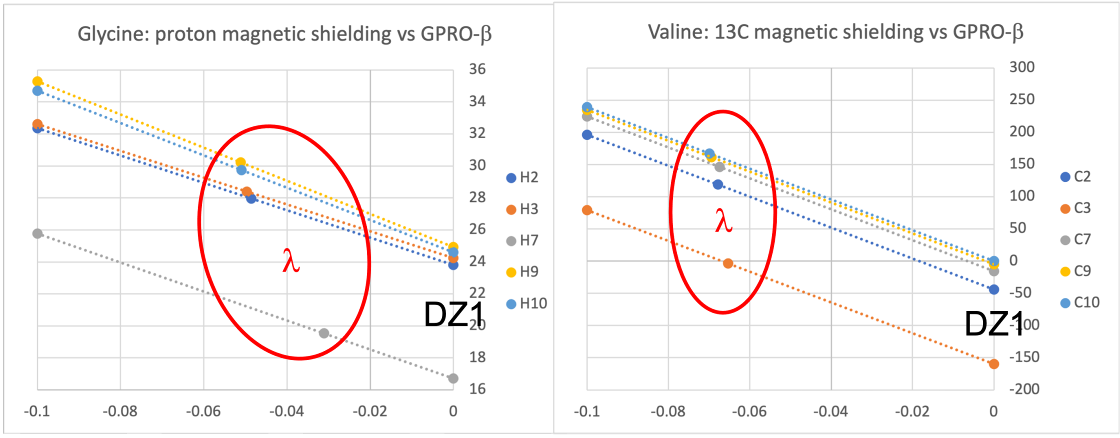

Figure S1: Application examples of the linear dependence of GPRO magnetic shieldings on β. Best β values that make 6-31+G(d,p) σ^N_{Av} perfectly equal to accurate pcSseg-4 forecasts λ, are located on lines connecting two calculated values placed on left and right vertical axis. The procedure is applied to all nuclei of all amino acids. Idem for GRRO-α

Table S1: Glycine, Cartesian coordinates and nuclear magnetic shielding constants.

Atm	x	y	z
C1	0.6889739	0.8394439	0.6776554
H2	−0.0782398	2.5814639	1.4798332
H3	2.6990692	0.6601836	1.1209410
C4	−0.6898478	−1.4362212	1.7954384
O5	−1.1354015	−1.7445309	4.0364584
O6	−1.3254452	−3.1276053	−0.0291466
H7	−0.7134479	−2.3894846	−1.6360985
N8	0.3471490	0.8528932	−2.1126480
H9	−1.3907856	1.6280978	−2.4873862
H10	1.5979756	2.1357595	−2.8450471

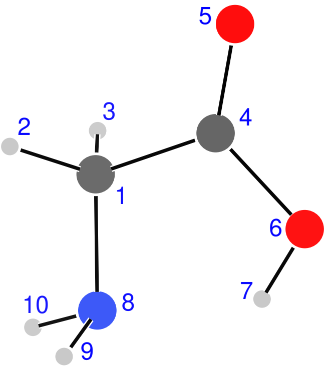

Atm	λ	GIAO	CSGT	DZ2	PZ2	GRRO $\bar{\alpha}$	GPRO $\bar{\beta}$	GRRO $\bar{\alpha}_{\mathrm{CE}}$	GPRO $\bar{\beta}_{\mathrm{CE}}$
C1	135.36	150.76	150.83	146.32	145.20	131.70	132.49	135.35	135.35
C4	−2.57	26.59	22.10	16.16	7.11	1.77	3.50	−3.36	−2.15
H10	29.74	30.14	28.82	25.78	28.74	28.86	30.90	29.30	30.82
H2	27.97	28.39	27.31	24.99	26.81	27.89	29.19	27.91	29.11
H3	28.40	28.73	27.70	25.41	27.31	28.32	29.51	28.33	29.43
H7	19.53	20.27	21.12	17.78	19.53	20.04	22.55	19.32	21.48
H9	30.22	30.66	29.24	26.16	29.42	29.31	31.42	29.76	31.31
N8	220.86	235.63	229.44	222.61	226.29	217.24	217.02	219.77	219.22
O5	−116.83	−68.57	−69.71	−81.69	−96.19	−102.08	−100.68	−115.16	−113.86
O6	110.85	141.37	132.17	121.44	117.95	101.48	101.27	111.09	110.65

Coordinates are in a.u. for the most stable conformer using MMFF94 force field
λ limit value in ppm obtained using the BHandHLYP/pcSseg-4 cobination of functional and basis set
Magnetic shieldings in ppm calculated at BHandHLY/P6-31+G(d,p) level of theory
For the definition of $\bar{\alpha}$, $\bar{\beta}$, $\bar{\alpha}_{\mathrm{CE}}$, and $\bar{\beta}_{\mathrm{CE}}$ see main text

Table S2: L-Alanine, Cartesian coordinates and nuclear magnetic shielding constants.

Atm	x	y	z
C1	−0.4214287	0.6913883	0.2571982
H2	−1.4573827	2.3633947	0.9013311
C3	−0.9924044	0.1359408	−2.5035760
H4	0.1098512	−1.4741037	−3.1979166
H5	−0.5159093	1.7641198	−3.6899615
H6	−3.0000050	−0.2834362	−2.7808379
C7	2.4065686	1.2278490	0.6602923
O8	3.7317714	2.5429294	−0.6925036
O9	3.2656602	0.0656598	2.7857583
H10	1.7951419	−0.8604134	3.4810202
N11	−1.1650926	−1.4890034	1.8869660
H12	−3.0998173	−1.5601351	1.9166343
H13	−0.6569534	−3.1241900	0.9755953

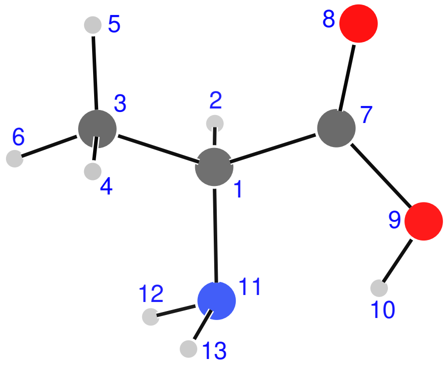

Atm	λ	GIAO	CSGT	DZ2	PZ2	GRRO $\bar{\alpha}$	GPRO $\bar{\beta}$	GRRO $\bar{\alpha}_{\rm CE}$	GPRO $\bar{\beta}_{\rm CE}$
C1	127.16	143.64	145.10	140.91	139.15	126.00	126.03	127.76	127.73
C3	159.70	173.36	174.01	169.88	170.88	155.79	155.08	161.23	160.58
C7	−5.48	23.78	19.69	14.02	5.23	−0.29	0.45	−5.42	−5.07
H10	19.30	20.05	20.82	17.66	19.41	19.87	21.68	19.16	20.72
H12	29.61	30.00	28.74	25.95	28.88	28.95	30.12	29.38	30.13
H13	30.42	30.83	29.38	26.56	29.70	29.59	30.77	30.03	30.76
H2	28.28	28.59	27.34	25.48	27.29	28.29	28.57	27.90	28.61
H4	30.50	30.76	29.02	26.67	29.26	29.73	30.24	30.25	30.90
H5	29.39	29.64	28.16	25.89	28.35	28.92	29.37	29.43	30.02
H6	30.45	30.82	29.05	26.71	29.26	29.82	30.17	30.34	30.86
N11	194.95	212.19	207.61	201.08	203.29	195.70	194.40	198.22	196.72
O8	−105.86	−58.74	−60.80	−72.46	−85.99	−92.77	−92.36	−105.84	−105.41
O9	107.17	137.38	128.30	117.77	114.22	97.83	96.85	107.44	106.32

See footnote of Table S1.

Table S3: L-Valine, Cartesian coordinates and nuclear magnetic shielding constants.

Atm	x	y	z
N1	−2.8763502	−2.4910176	−0.2209667
C2	−1.3534525	−0.4773903	1.0616838
C3	−2.5693403	2.0969648	0.4344668
O4	−2.1627483	4.0778377	1.5412808
H5	−1.9001132	−4.1565186	−0.0836568
H6	−1.5366701	−0.7792951	3.1030387
C7	1.4487034	−0.6279850	0.2599106
H8	2.1145540	−2.5518296	0.6707860
C9	3.1065380	1.1640144	1.8149990
C10	1.8736544	−0.1602985	−2.5662230
H11	1.3259346	1.7584350	−3.1134350
H12	0.7972243	−1.4939731	−3.7238823
H13	3.8732901	−0.4003981	−3.0462179
H14	2.7196291	3.1480838	1.3788655
H15	5.1097758	0.8206161	1.4207013
H16	2.8086489	0.8734251	3.8419094
O17	−4.1762157	1.8955606	−1.5615674
H18	−4.1346876	0.0917595	−2.0591147
H19	−4.4683746	−2.7879910	0.8474217

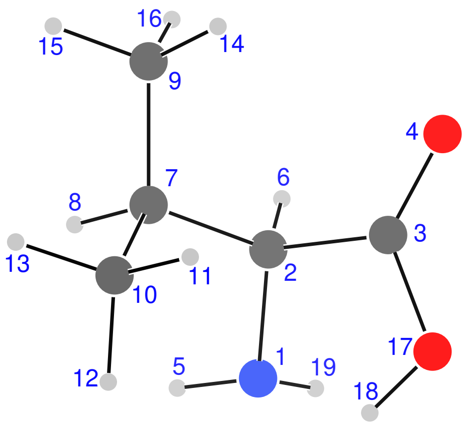

Atm	λ	GIAO	CSGT	DZ2	PZ2	GRRO $\bar{\alpha}$	GPRO $\bar{\beta}$	GRRO $\bar{\alpha}_{\rm CE}$	GPRO $\bar{\beta}_{\rm CE}$
C10	167.21	180.25	180.24	176.32	177.67	162.41	161.01	167.87	166.56
C2	119.00	135.90	137.34	133.38	131.05	118.68	117.87	120.43	119.57
C3	−3.56	25.48	21.11	15.66	7.11	1.49	1.52	−3.62	−3.96
C7	146.48	161.88	165.58	161.90	161.23	147.22	146.17	147.10	146.46
C9	161.63	175.07	175.89	171.92	173.04	157.96	156.61	163.41	162.16
H11	30.91	31.06	29.19	27.07	29.64	30.13	30.11	30.65	30.75
H12	30.25	30.43	28.75	26.63	29.07	29.70	29.55	30.22	30.20
H13	30.66	30.90	29.11	26.89	29.44	30.01	29.91	30.55	30.59
H14	30.07	30.23	28.60	26.39	28.95	29.44	29.45	29.95	30.07
H15	30.60	30.84	29.08	26.82	29.42	29.97	29.87	30.51	30.55
H16	30.45	30.70	28.96	26.73	29.28	29.84	29.76	30.36	30.41
H18	19.53	20.25	20.82	17.86	19.45	20.06	21.33	19.36	20.47
H19	29.82	30.25	28.97	26.37	29.19	29.34	29.97	29.77	29.97
H5	30.17	30.59	29.30	26.70	29.51	29.63	30.19	30.05	30.21
H6	27.98	28.39	27.14	25.47	27.11	28.24	28.03	27.86	28.07
H8	30.01	30.28	28.47	26.71	28.75	29.66	29.23	29.95	29.98
N1	203.21	219.61	214.18	207.89	210.19	202.55	200.51	205.08	202.85
O17	103.47	133.67	124.65	114.37	110.31	94.48	92.85	104.10	102.38
O4	−120.56	−72.54	−74.98	−86.36	−101.29	−106.57	−106.95	−119.64	−119.96

See footnote of Table S1.

Table S4: L-Leucine, Cartesian coordinates and nuclear magnetic shielding constants.

Atm	x	y	z
N1	−0.6308864	−0.0542525	−4.0211066
C2	−2.0226658	0.4082912	−1.6080554
C3	−3.9993095	2.4954518	−2.0919673
O4	−5.1671597	3.6449371	−0.4699078
H5	0.2848761	−1.7514920	−3.8548818
H6	−3.0949635	−1.2990830	−1.1414808
C7	−0.2495564	1.1774816	0.5547041
C8	0.7333410	−3.3037470	2.0376840
H9	0.6472753	2.9895214	0.0793425
C10	1.8259119	−0.7549021	1.2256379
H11	3.0351882	−1.0624256	−0.4288926
H12	−1.3900727	1.5524744	2.2485915
C13	3.5011764	0.3051936	3.3338776
H14	4.3596298	2.0985011	2.7592843
H15	5.0346541	−1.0092323	3.7858268
H16	2.4136571	0.6389325	5.0632399
H17	−0.2783032	−4.2192301	0.4842451
H18	−0.5783045	−3.0865988	3.6238872
H19	2.2409973	−4.5931314	2.6289958
O20	−4.3285656	2.8805496	−4.6118793
H21	−3.1432253	1.7005065	−5.4532975
H22	0.8063052	1.2422539	−4.1438476

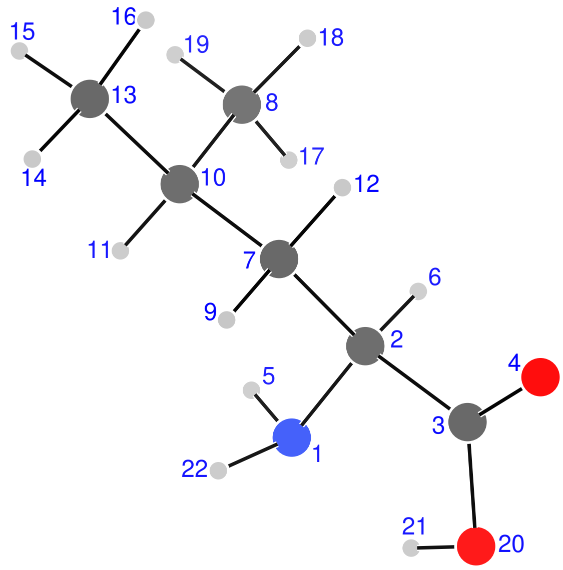

Atm	λ	GIAO	CSGT	DZ2	PZ2	GRRO $\bar{\alpha}$	GPRO $\bar{\beta}$	GRRO $\bar{\alpha}_{\mathrm{CE}}$	GPRO $\bar{\beta}_{\mathrm{CE}}$
C10	154.78	169.89	173.68	170.06	170.00	155.41	154.13	155.29	154.47
C13	158.37	171.98	172.74	168.79	169.87	154.89	153.33	160.35	158.92
C2	124.98	141.01	142.72	138.72	136.74	123.95	123.03	125.69	124.67
C3	−6.14	23.17	19.05	13.56	4.79	−0.69	−0.79	−5.82	−6.33
C7	137.37	152.98	155.22	151.45	150.51	137.07	135.57	140.50	139.46
C8	163.60	176.68	177.48	173.51	174.79	159.62	158.08	165.08	163.66
H11	30.24	30.50	28.56	26.84	28.83	29.81	29.07	30.09	29.81
H12	29.51	29.73	28.27	26.41	28.50	29.28	28.97	29.27	29.31
H14	30.54	30.78	29.04	26.79	29.39	29.93	29.65	30.46	30.33
H15	30.41	30.64	28.84	26.59	29.19	29.75	29.50	30.29	30.19
H16	30.68	30.87	28.94	26.70	29.37	29.83	29.66	30.37	30.36
H17	30.55	30.83	28.99	26.73	29.22	29.86	29.56	30.39	30.22
H18	30.98	31.15	29.15	26.91	29.60	30.03	29.93	30.56	30.62
H19	30.50	30.74	28.88	26.63	29.20	29.77	29.53	30.31	30.22
H21	18.93	19.69	20.46	17.41	18.96	19.57	20.79	18.87	19.86
H22	30.82	31.23	29.89	27.27	30.19	30.24	30.67	30.66	30.65
H5	29.33	29.76	28.56	26.01	28.62	28.91	29.25	29.33	29.23
H6	28.32	28.63	27.47	25.79	27.48	28.53	28.12	28.15	28.12
H9	30.35	30.64	29.08	27.16	29.30	30.06	29.74	30.05	30.08
N1	207.95	223.82	218.75	212.46	214.93	207.06	204.91	209.57	207.20
O20	106.63	136.85	127.92	117.54	113.85	97.61	95.96	107.22	105.44
O4	−102.43	−54.83	−57.90	−69.33	−83.00	−89.61	−90.06	−102.68	−103.12

See footnote of Table S1.

Table S5: L-Isoleucine, Cartesian coordinates and nuclear magnetic shielding constants.

Atm	x	y	z
N1	1.9943416	−3.8282988	−0.1002061
C2	0.7575576	−1.4947664	0.9292246
C3	−1.7525720	−2.2919018	2.1777417
O4	−2.9941932	−1.0067733	3.6340643
H5	3.4845328	−3.2744536	−1.2041222
H6	2.0049287	−0.7805940	2.4172234
C7	0.3840174	0.5016020	−1.1743463
C8	−1.3027543	−0.4482136	−3.3388776
H9	2.2519187	0.8904730	−1.9973124
C10	−0.6552861	3.0528553	−0.2114345
H11	−2.5868347	2.8335822	0.5024626
H12	−0.7794874	4.3646379	−1.8135520
C13	0.9710879	4.2886520	1.8130247
H14	0.9278385	3.2205629	3.5817994
H15	0.2668658	6.1907810	2.2216534
H16	2.9361617	4.4551191	1.1899548
H17	−0.5559965	−2.1834342	−4.1805515
H18	−1.4105521	0.9652030	−4.8480785
H19	−3.2323615	−0.8214756	−2.6920150
O20	−2.4448137	−4.6519891	1.4311706
H21	−1.0920610	−5.2293381	0.2736679
H22	2.8276619	−4.7522298	1.3885083

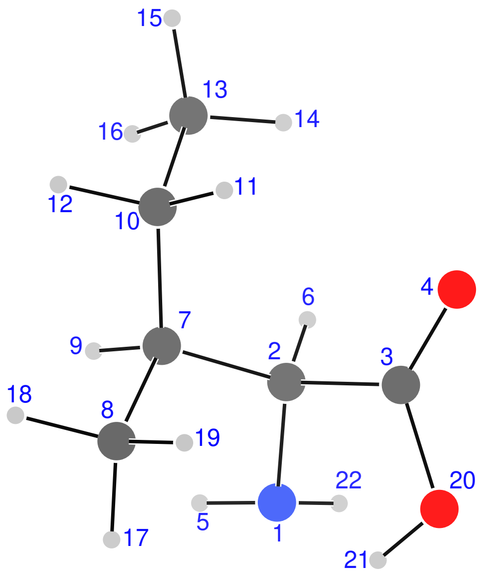

Atm	λ	GIAO	CSGT	DZ2	PZ2	GRRO $\bar{\alpha}$	GPRO $\bar{\beta}$	GRRO $\bar{\alpha}_{\mathrm{CE}}$	GPRO $\bar{\beta}_{\mathrm{CE}}$
C10	152.82	167.53	169.57	165.79	165.96	151.45	150.18	154.90	154.18
C13	169.81	182.46	182.95	178.93	180.71	165.01	163.66	170.46	169.24
C2	124.08	140.49	141.60	137.69	135.84	123.03	122.10	124.77	123.80
C3	−3.61	25.56	21.15	15.75	7.28	1.63	1.53	−3.49	−3.93
C7	139.68	155.56	159.57	155.95	155.09	141.34	140.12	141.21	140.38
C8	166.86	179.85	179.74	175.86	177.29	161.98	160.45	167.43	165.97
H11	29.78	29.94	28.24	26.28	28.55	29.26	29.11	29.26	29.52
H12	30.25	30.49	28.73	26.72	29.04	29.81	29.57	29.81	30.01
H14	30.07	30.30	28.54	26.30	28.93	29.43	29.30	29.96	29.97
H15	30.40	30.62	28.83	26.53	29.20	29.72	29.58	30.26	30.28
H16	31.01	31.25	29.34	27.02	29.80	30.20	30.05	30.74	30.74
H17	30.23	30.42	28.80	26.72	29.20	29.76	29.53	30.28	30.16
H18	30.71	30.98	29.27	27.07	29.67	30.17	30.02	30.69	30.68
H19	30.85	31.03	29.25	27.17	29.75	30.21	30.11	30.72	30.73
H21	19.50	20.23	20.77	17.87	19.57	20.05	21.24	19.36	20.40
H22	29.83	30.26	28.97	26.41	29.25	29.37	29.90	29.80	29.90
H5	30.27	30.68	29.34	26.79	29.61	29.71	30.21	30.13	30.23
H6	27.64	28.07	26.78	25.14	26.86	27.92	27.57	27.53	27.63
H9	30.22	30.48	28.69	27.01	29.03	29.92	29.44	30.18	30.15
N1	204.84	221.01	215.49	209.26	211.66	203.93	201.78	206.45	204.12
O20	102.23	132.65	123.51	113.29	109.26	93.41	91.68	103.03	101.22
O4	−117.13	−69.49	−72.29	−83.58	−98.10	−103.78	−104.26	−116.84	−117.25

See footnote of Table S1.

Table S6: L-Proline, Cartesian coordinates and nuclear magnetic shielding constants.

Atm	x	y	z
N1	−0.4111642	−0.5677479	2.1732882
C2	−0.6460414	−1.6280759	−0.4435607
C3	1.5397487	−3.4761792	−0.9003105
O4	1.8143559	−4.7599039	−2.7974967
C5	−0.8897482	2.1543701	1.9427008
C6	−0.5081210	0.6140952	−2.2473018
H7	0.8144331	0.3600858	−3.8171507
C8	0.2589979	2.8244311	−0.5878757
H9	−2.3873525	0.9792257	−3.0420413
H10	−0.4316680	4.6296931	−1.3203092
H11	2.3244770	2.9264121	−0.4454240
H12	−0.0248954	3.2042586	3.4979330
H13	−2.9283680	2.5237968	1.9443476
H14	−2.4270876	−2.6663680	−0.6274879
O15	3.1822532	−3.4998558	1.0734351
H16	2.4990856	−2.2639022	2.3025801
H17	−1.7789053	−1.3543355	3.2946736

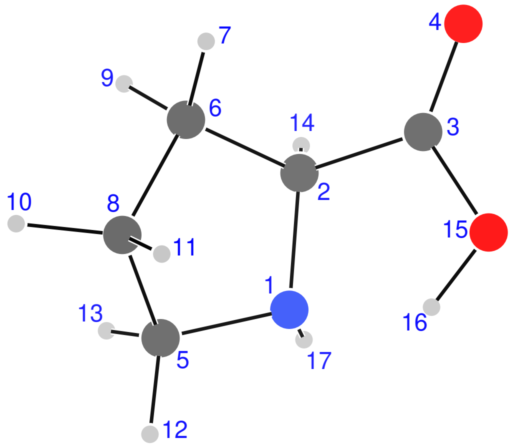

Atm	λ	GIAO	CSGT	DZ2	PZ2	GRRO $\bar{\alpha}$	GPRO $\bar{\beta}$	GRRO $\bar{\alpha}_{\mathrm{CE}}$	GPRO $\bar{\beta}_{\mathrm{CE}}$
C2	120.10	136.67	137.08	133.13	130.66	118.37	117.42	120.07	119.05
C3	−7.28	21.84	17.55	11.98	3.08	−2.31	−2.09	−7.45	−7.63
C5	134.43	150.40	150.34	146.36	144.99	132.09	130.88	135.71	133.92
C6	154.22	168.51	169.34	165.55	165.32	151.01	149.75	154.40	153.58
C8	155.94	170.32	170.91	167.09	167.00	152.56	151.34	155.97	155.20
H10	29.79	30.06	28.72	26.79	28.77	29.73	29.54	29.70	29.89
H11	29.85	30.02	28.47	26.51	28.47	29.44	29.35	29.42	29.71
H12	28.45	28.66	27.48	25.51	27.34	28.47	28.41	28.48	28.46
H13	28.89	29.14	27.78	25.81	27.69	28.80	28.67	28.80	28.70
H14	27.86	28.26	27.40	25.83	27.32	28.50	28.30	28.09	28.30
H16	18.62	19.30	19.90	16.97	18.41	19.06	20.33	18.36	19.48
H17	29.24	29.69	28.57	26.38	28.38	29.16	29.39	29.25	29.37
H7	29.37	29.64	28.44	26.63	28.49	29.42	29.31	29.39	29.61
H9	29.50	29.78	28.50	26.59	28.52	29.49	29.36	29.46	29.69
N1	187.93	205.67	202.23	196.17	196.56	190.17	187.97	188.75	187.49
O15	101.04	130.75	123.03	112.71	108.58	92.77	91.23	102.37	100.72
O4	−97.27	−51.00	−53.85	−65.51	−78.98	−85.84	−85.70	−98.92	−98.78

See footnote of Table S1.

Table S7: L-Serine, Cartesian coordinates and nuclear magnetic shielding constants.

Atm	x	y	z
N1	−2.0032257	−1.9140931	0.6467710
C2	−0.5034413	0.4722091	0.9871489
C3	−1.5229402	2.4683874	−0.8529916
O4	−0.5386200	4.5089233	−1.2916249
H5	−1.0887172	−2.8488935	−0.8097579
H6	−0.8339359	1.2153916	2.8908083
C7	2.3228377	0.0236974	0.5203976
08	2.6414785	−1.8006219	−1.4571181
H9	3.2033098	−0.7653710	2.2182290
H10	3.3196067	1.7563328	−0.0098992
H11	4.4276515	−1.7879067	−1.9075403
O12	−3.7494855	1.7184737	−1.8951610
H13	−4.0664261	0.0063825	−1.2000534
H14	−1.6080923	−3.0529117	2.1607916

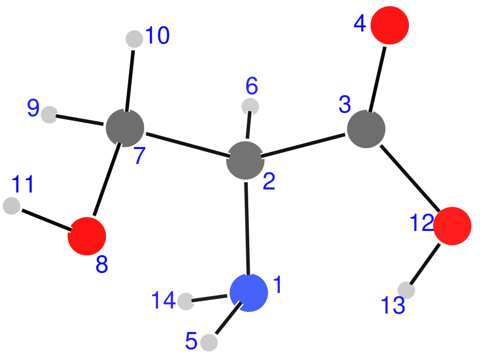

Atm	λ	GIAO	CSGT	DZ2	PZ2	GRRO $\bar{\alpha}$	GPRO $\bar{\beta}$	GRRO $\bar{\alpha}_{\mathrm{CE}}$	GPRO $\bar{\beta}_{\mathrm{CE}}$
C2	124.19	139.99	141.31	137.06	135.28	122.19	122.29	123.92	123.73
C3	−6.32	22.96	18.79	13.14	4.72	−1.13	−0.35	−6.26	−6.03
C7	119.61	136.32	136.60	132.05	130.31	117.97	118.10	120.53	119.07
H10	26.97	27.33	26.34	24.02	26.21	26.94	27.86	26.78	27.36
H11	30.12	30.60	29.81	26.37	29.66	29.05	31.61	29.60	30.81
H13	19.01	19.72	20.65	17.41	19.28	19.62	21.42	18.91	20.37
H14	30.43	30.76	29.55	26.77	29.85	29.75	30.88	30.17	30.77
H5	28.02	28.39	27.44	24.50	27.78	27.40	28.53	27.80	28.38
H6	28.26	28.66	27.73	25.90	27.78	28.63	29.03	28.24	28.89
H9	27.96	28.28	27.09	24.68	26.60	27.74	28.64	27.57	28.11
N1	224.39	238.65	232.66	226.10	229.88	220.78	219.37	223.29	221.55
O12	100.75	131.36	122.41	111.88	108.18	91.96	91.00	101.57	100.38
O4	−101.91	−54.60	−56.42	−68.03	−81.43	−88.34	−87.90	−101.41	−101.08
O8	309.13	318.37	301.80	291.21	299.07	271.72	271.32	310.53	307.40

See footnote of Table S1.

Table S8: L-Threonine, Cartesian coordinates and nuclear magnetic shielding constants.

Atm	x	y	z
N1	2.8830239	−1.3494884	0.3440470
C2	0.5037012	−0.1796615	1.3693012
C3	−0.8066700	−2.1281518	3.0757754
O4	−2.9217776	−1.9118148	3.9729724
H5	3.9554515	0.0854452	−0.3868299
H6	1.0174023	1.4023719	2.6035168
C7	−1.2991489	0.6562687	−0.7649891
O8	−1.1503187	−1.1517655	−2.7889845
C9	−0.5609645	3.2241973	−1.8399585
H10	−3.2622835	0.7137603	−0.1101829
H11	−2.5537677	−0.7999644	−3.9297312
H12	−0.6280074	4.6923302	−0.3856392
H13	−1.8478771	3.7591574	−3.3707654
H14	1.3398964	3.1807806	−2.6579537
O15	0.7355829	−4.1272373	3.5565623
H16	2.3018665	−3.7830725	2.5851249
H17	2.2938907	−2.2831553	−1.2722657

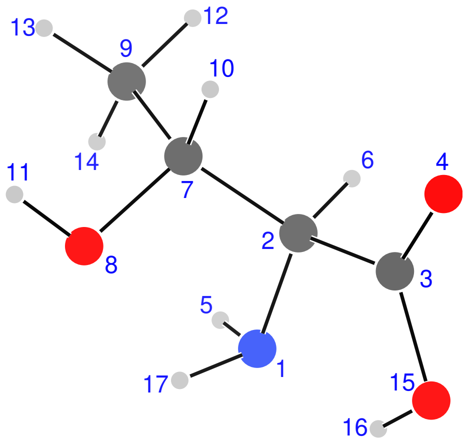

Atm	λ	GIAO	CSGT	DZ2	PZ2	GRRO $\bar{\alpha}$	GPRO $\bar{\beta}$	GRRO $\bar{\alpha}_{\mathrm{CE}}$	GPRO $\bar{\beta}_{\mathrm{CE}}$
C2	119.56	135.73	137.23	133.14	130.83	118.39	117.82	120.13	119.42
C3	−6.19	22.93	18.88	13.36	4.58	−0.89	−0.66	−6.01	−6.22
C7	114.23	131.64	132.96	128.77	126.19	114.39	113.86	114.98	114.17
C9	157.71	171.22	172.01	167.92	168.74	153.88	152.79	159.32	158.29
H10	26.85	27.17	26.18	24.33	25.89	27.11	27.19	26.65	27.13
H11	30.06	30.52	29.71	26.55	29.54	29.16	30.90	29.60	30.59
H12	30.50	30.80	29.26	26.97	29.45	30.01	30.13	30.53	30.79
H13	30.41	30.70	29.02	26.68	29.15	29.76	29.92	30.29	30.60
H14	30.26	30.52	28.73	26.47	28.92	29.52	29.64	30.03	30.28
H16	18.98	19.70	20.60	17.44	19.08	19.63	21.09	18.92	20.13
H17	28.32	28.68	27.70	24.89	27.72	27.75	28.44	28.15	28.42
H5	30.10	30.44	29.25	26.57	29.35	29.51	30.23	29.93	30.22
H6	28.51	28.87	27.90	26.21	27.91	28.90	28.82	28.51	28.82
N1	230.93	244.53	238.71	232.30	235.92	226.98	225.08	229.49	227.37
O15	101.10	132.00	123.07	112.63	108.72	92.71	91.35	102.33	100.81
O4	−99.59	−52.54	−54.78	−66.24	−79.70	−86.53	−86.67	−99.60	−99.74
O8	270.81	284.13	270.32	260.13	264.82	240.71	239.23	272.12	269.76

See footnote of Table S1.

Table S9: L-Asparagine, Cartesian coordinates and nuclear magnetic shielding constants.

Atm	x	y	z
N1	−2.6565368	1.7273986	−2.5286288
C2	−0.5581934	0.2852896	−1.2837358
C3	−1.4811761	−2.4568884	−0.9421998
O4	−0.5519128	−4.0141428	0.4876132
H5	−1.9156674	3.4085574	−3.1489096
H6	1.0439119	0.2329108	−2.5948694
C7	0.1416418	1.4280499	1.2700984
H8	−1.0352596	0.6924952	2.8086040
H9	−0.0980595	3.4878908	1.2481031
C10	2.8902686	0.9349526	1.8665485
O11	4.5111793	2.5837618	1.7263979
N12	3.4808778	−1.4648257	2.5623212
H13	2.2089284	−2.9128937	2.5281613
H14	5.2980409	−1.8784051	2.9869345
O15	−3.4864238	−2.9578571	−2.4631650
H16	−3.8610371	−1.3549611	−3.3577569
H17	−3.9305820	2.2586672	−1.1655169

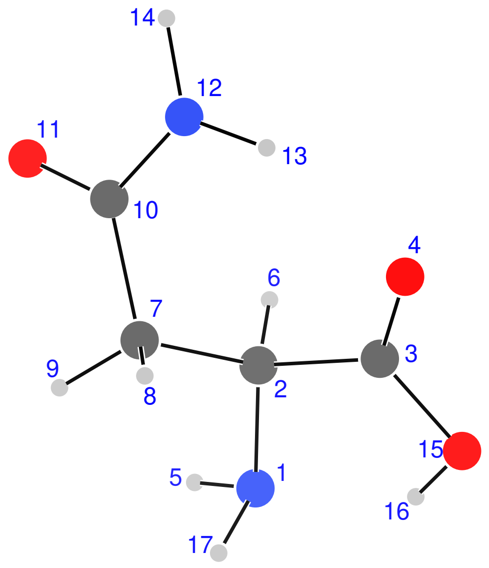

Atm	λ	GIAO	CSGT	DZ2	PZ2	GRRO $\bar{\alpha}$	GPRO $\bar{\beta}$	GRRO $\bar{\alpha}_{\mathrm{CE}}$	GPRO $\bar{\beta}_{\mathrm{CE}}$
C10	0.49	28.88	26.39	20.76	12.06	6.35	6.47	0.54	0.08
C2	125.54	141.87	142.59	138.53	136.45	123.67	123.08	125.40	124.62
C3	−10.86	18.89	14.78	9.46	0.24	−4.77	−4.87	−9.91	−10.45
C7	137.70	152.95	152.71	148.68	147.39	134.04	132.92	137.44	136.59
H13	22.45	23.10	23.66	20.29	22.48	22.93	23.98	22.76	23.39
H14	26.54	26.89	26.31	23.18	25.58	26.04	27.10	25.85	26.40
H16	18.92	19.71	20.42	17.44	18.97	19.57	20.94	18.87	19.98
H17	30.19	30.61	29.41	26.71	29.60	29.66	30.35	30.08	30.26
H5	29.27	29.64	28.52	25.87	28.53	28.76	29.51	29.18	29.44
H6	28.16	28.53	27.41	25.70	27.33	28.37	28.37	27.97	28.29
H8	28.62	29.02	28.02	26.11	27.93	28.80	28.75	28.77	28.94
H9	29.34	29.77	28.80	26.86	28.63	29.55	29.52	29.52	29.71
N1	191.62	208.86	204.16	197.77	199.49	192.34	190.53	194.85	192.77
N12	140.14	162.77	157.22	149.96	149.23	143.96	142.77	140.91	138.95
O11	−92.75	−46.96	−48.37	−60.12	−73.22	−80.55	−80.45	−92.69	−93.12
O15	99.02	129.05	121.32	111.04	106.87	91.10	89.56	100.71	99.00
O4	−90.98	−44.94	−46.74	−57.81	−70.97	−78.10	−79.01	−91.16	−92.08

See footnote of Table S1.

Table S10: L-Glutamine, Cartesian coordinates and nuclear magnetic shielding constants.

Atm	x	y	z
N1	2.5848826	2.6837929	2.7151372
C2	1.0282766	1.3348614	0.7776231
C3	−1.2542350	3.0192267	0.1303032
O4	−2.6480443	2.7213820	−1.6875910
H5	3.6737429	3.9929212	1.7839443
H6	2.1931294	1.1192382	−0.9180767
C7	0.1813553	−1.2267132	1.8396124
H8	−0.8707299	−0.9440823	3.6057157
H9	1.8645851	−2.3399289	2.3239956
C10	−1.4728550	−2.8006611	0.0652330
H11	−3.3226898	−1.9129599	−0.2050393
H12	−1.8410009	−4.6523147	0.9192960
C13	−0.2110365	−3.2472304	−2.4548394
O14	1.4781285	−4.7974217	−2.7700231
N15	−1.0054773	−1.7074446	−4.3552152
H16	−2.2706576	−0.2818747	−4.0792351
H17	−0.2365964	−1.9147761	−6.0918035
O18	−1.5706129	4.8747861	1.8725703
H19	−0.1811465	4.6517913	3.1089000
H20	3.8809817	1.4274079	3.4194927

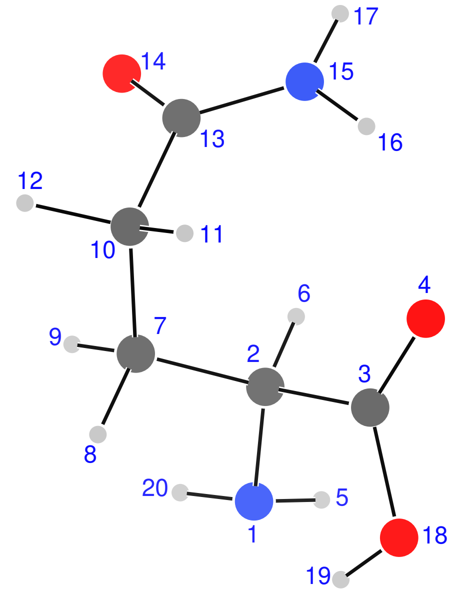

Atm	λ	GIAO	CSGT	DZ2	PZ2	GRRO $\bar{\alpha}$	GPRO $\bar{\beta}$	GRRO $\bar{\alpha}_{\mathrm{CE}}$	GPRO $\bar{\beta}_{\mathrm{CE}}$
C10	150.51	164.89	165.39	161.35	160.96	146.73	145.81	150.13	149.55
C13	−2.10	26.76	24.38	18.79	10.26	4.47	4.67	−1.34	−1.69
C2	125.99	142.64	143.79	139.83	137.89	125.03	124.45	126.75	126.04
C3	−8.09	21.42	17.46	12.18	3.18	−1.97	−2.07	−7.10	−7.61
C7	150.94	165.30	167.43	163.53	163.14	148.98	148.06	152.38	151.83
H11	29.15	29.51	28.50	26.51	28.48	29.31	29.37	29.28	29.60
H12	29.45	29.75	28.69	26.75	28.68	29.53	29.59	29.50	29.84
H16	23.49	24.08	24.33	21.22	23.48	23.89	24.88	23.72	24.32
H17	26.55	26.92	26.26	23.27	25.68	26.11	27.14	25.92	26.48
H19	19.11	19.86	20.42	17.51	19.03	19.65	21.01	18.95	20.11
H20	29.94	30.37	29.12	26.50	29.26	29.41	30.23	29.82	30.19
H5	29.80	30.21	28.95	26.35	29.23	29.31	30.07	29.74	30.02
H6	27.73	28.16	26.82	25.26	26.82	27.98	27.89	27.58	27.86
H8	29.91	30.16	28.74	26.81	28.81	29.65	29.81	29.63	30.09
H9	29.53	29.85	28.49	26.51	28.49	29.38	29.49	29.35	29.76
N1	204.76	220.90	216.16	209.86	212.31	204.50	202.66	207.01	204.94
N15	135.06	158.10	152.62	145.54	144.54	139.57	138.35	136.53	134.57
O14	−80.04	−34.11	−35.43	−47.15	−59.64	−67.54	−67.37	−79.68	−80.01
O18	108.93	138.74	129.86	119.65	115.84	99.73	98.14	109.34	107.62
O4	−102.73	−54.93	−58.12	−69.17	−82.95	−89.45	−90.28	−102.52	−103.32

See footnote of Table S1.

Table S11: L-Cysteine, Cartesian coordinates and nuclear magnetic shielding constants.

Atm	x	y	z
N1	2.7415870	−0.2084054	0.4237238
C2	0.1437950	0.6203176	−0.3562179
C3	−0.7251036	2.7059923	1.4619422
O4	−2.8929940	3.4552602	1.6967584
H5	3.4077414	−1.3455355	−1.0025635
H6	0.2923682	1.4942636	−2.2291637
C7	−1.8018424	−1.5273005	−0.3649180
S8	−0.8512508	−4.1122565	−2.4408943
H9	−1.1842823	−2.8733111	−4.6287399
H10	−2.0501363	−2.2932814	1.5430867
H11	−3.6486559	−0.8384653	−0.9961420
O12	1.2581266	3.6724252	2.7774156
H13	2.7650623	2.7098087	2.2195228
H14	2.5455847	−1.4595118	1.8961897

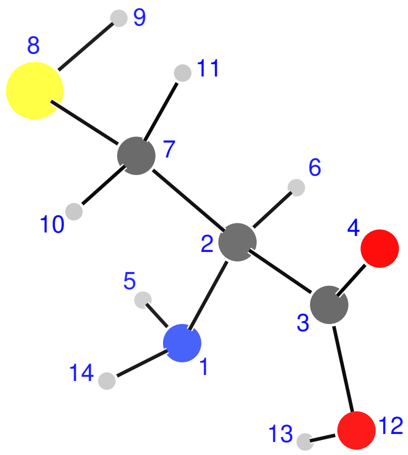

Atm	λ	GIAO	CSGT	DZ2	PZ2	GRRO $\bar{\alpha}$	GPRO $\bar{\beta}$	GRRO $\bar{\alpha}_{\mathrm{CE}}$	GPRO $\bar{\beta}_{\mathrm{CE}}$
C2	118.15	135.10	137.34	132.57	130.18	117.64	117.95	119.42	119.62
C3	−4.62	24.73	20.76	14.86	6.14	0.55	1.24	−4.57	−4.30
C7	147.11	161.93	165.19	159.46	159.35	144.92	145.64	147.44	147.13
H10	29.77	29.96	28.60	25.54	27.73	28.48	29.41	29.31	29.96
H11	27.60	27.98	26.69	23.61	25.62	26.54	27.48	27.36	28.04
H13	19.24	20.03	20.87	17.59	19.27	19.76	21.57	19.07	20.61
H14	30.89	31.22	29.82	26.85	29.77	29.87	31.11	30.32	31.10
H5	27.99	28.46	27.38	24.58	26.79	27.47	28.59	27.91	28.61
H6	28.63	29.03	27.90	25.59	27.46	28.32	28.89	27.94	28.92
H9	30.02	30.37	29.91	23.56	27.80	27.02	30.09	29.21	30.89
N1	205.37	221.26	216.20	209.50	211.86	204.14	202.88	206.68	205.19
O12	101.45	131.78	123.58	112.95	109.09	92.99	91.93	102.62	101.40
O4	−107.82	−60.24	−62.61	−74.40	−88.14	−94.71	−94.48	−107.77	−107.54
S8	583.29	639.44	574.25	542.26	545.96	572.85	570.50	585.06	581.34

See footnote of Table S1.

Table S12: L-Methionine, Cartesian coordinates and nuclear magnetic shielding constants.

Atm	x	y	z
N1	0.6909115	3.0029064	2.6216296
C2	1.4434285	2.9559015	−0.0948642
C3	−0.8314789	3.7849750	−1.7033115
O4	−0.9902927	3.5846272	−3.9957321
H5	−0.1494245	1.2869533	3.0208150
H6	2.8830368	4.4259700	−0.3412912
C7	2.5213883	0.4070002	−0.9689033
C8	0.6615598	−1.8073801	−1.1607174
H9	4.1239808	−0.1207580	0.2388943
H10	3.3206229	0.6867987	−2.8645575
H11	1.6232349	−3.3956128	−2.0760877
H12	−0.9675147	−1.3049632	−2.3297073
S13	−0.4469318	−2.8941683	1.9137537
C14	−2.5463529	−5.3765452	0.8461547
H15	−3.4135198	−6.2850739	2.4863216
H16	−1.4931210	−6.8017078	−0.2150014
H17	−4.0446389	−4.5899813	−0.3383374
O18	−2.6719386	4.8115642	−0.2365706
H19	−2.0256911	4.7010386	1.5197138
H20	2.3127414	2.9284555	3.6777989

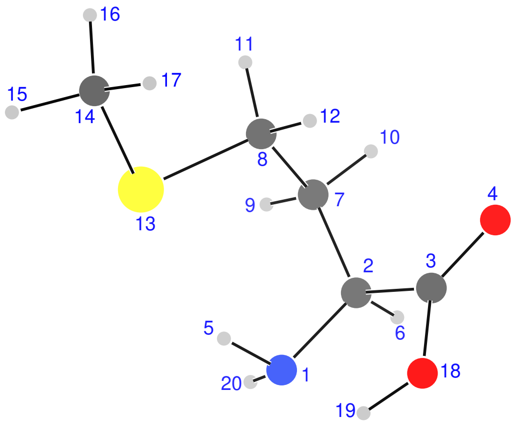

Atm	λ	GIAO	CSGT	DZ2	PZ2	GRRO $\bar{\alpha}$	GPRO $\bar{\beta}$	GRRO $\bar{\alpha}_{\mathrm{CE}}$	GPRO $\bar{\beta}_{\mathrm{CE}}$
C14	162.40	175.85	178.23	172.36	174.75	158.45	158.90	164.10	162.87
C2	122.06	138.79	140.88	136.63	134.58	121.77	121.46	123.51	123.11
C3	−5.78	23.68	19.52	13.87	4.99	−0.38	0.03	−5.50	−5.48
C7	150.18	165.10	167.26	162.96	162.82	148.42	147.79	151.87	151.68
C8	147.49	162.93	166.67	161.31	161.81	146.87	147.08	149.35	148.63
H10	28.86	29.18	27.80	25.61	27.67	28.49	28.73	28.49	29.09
H11	28.91	29.28	27.53	24.58	26.93	27.69	28.34	28.54	29.00
H12	28.84	29.18	27.43	24.53	26.90	27.56	28.23	28.38	28.82
H15	29.57	29.85	28.26	25.00	27.97	28.30	29.00	29.48	29.73
H16	29.57	29.93	28.03	24.58	27.52	27.97	28.92	29.18	29.67
H17	29.36	29.71	27.85	24.49	27.44	27.85	28.80	29.04	29.55
H19	18.84	19.62	20.43	17.34	18.90	19.50	21.09	18.81	20.18
H20	30.01	30.39	29.30	26.63	29.38	29.56	30.40	29.98	30.40
H5	27.60	28.24	27.79	24.94	27.45	27.76	28.43	28.18	28.46
H6	28.12	28.49	27.43	25.59	27.31	28.34	28.46	27.95	28.47
H9	29.95	30.30	28.65	26.48	28.65	29.40	29.54	29.40	29.91
N1	198.61	215.51	211.47	205.03	207.16	199.65	197.87	202.17	200.19
O18	98.57	128.73	120.70	110.28	106.04	90.37	89.08	99.98	98.58
O4	−102.60	−55.97	−58.15	−69.73	−83.56	−90.00	−90.02	−103.06	−103.05
S13	541.56	605.24	554.46	524.23	524.42	553.83	550.27	542.23	539.99

See footnote of Table S1.

Table S13: L-Phenylalanine, Cartesian coordinates and nuclear magnetic shielding constants.

Atm	x	y	z
N1	−0.4107556	−4.1160342	0.6988758
C2	−2.5306350	−2.2404932	0.5065583
C3	−4.9142217	−3.6606628	−0.3498511
O4	−6.8501432	−2.6856568	−1.1358427
H5	1.0162084	−3.2362576	1.6788775
H6	−2.9151486	−1.5360279	2.4163141
C7	−1.9640176	−0.0621847	−1.3285345
C8	0.3297627	1.4659206	−0.5801570
H9	−3.5920303	1.2204737	−1.4350459
H10	−1.7258613	−0.8001178	−3.2540584
C11	4.6039063	4.2490168	0.8561364
C12	2.5775441	1.2890343	−1.9700243
C13	0.2459637	3.0654006	1.5299842
C14	2.3752810	4.4476372	2.2440615
C15	4.7041828	2.6738543	−1.2515219
H16	2.6908473	0.0812534	−3.6293950
H17	−1.4821804	3.2571935	2.6276841
H18	2.2928029	5.6884279	3.8794202
H19	6.4391565	2.5324481	−2.3426430
H20	6.2589575	5.3324959	1.4096377
O21	−4.6187351	−6.1995645	−0.0795664
H22	0.3503756	−4.3310676	−1.0719152
H23	−2.8812600	−6.4350892	0.5810056

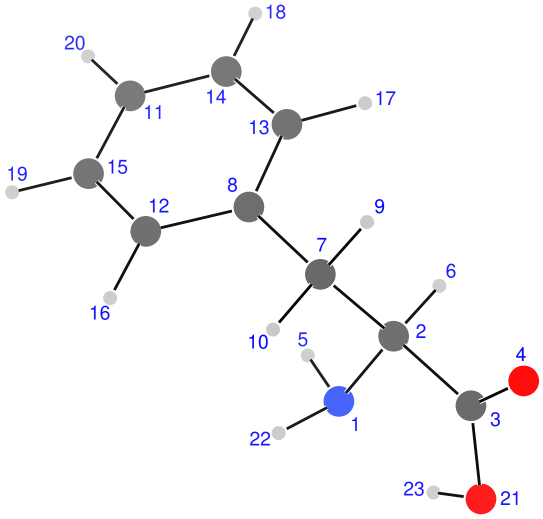

Atm	λ	GIAO	CSGT	DZ2	PZ2	GRRO $\bar{\alpha}$	GPRO $\bar{\beta}$	GRRO $\bar{\alpha}_{\text{CE}}$	GPRO $\bar{\beta}_{\text{CE}}$
C11	45.69	71.27	68.64	63.67	57.43	48.84	47.43	46.95	46.31
C12	42.45	67.95	65.34	60.56	54.42	45.80	44.10	43.92	42.97
C13	44.85	70.48	67.71	62.90	56.83	48.14	46.48	46.26	45.34
C14	41.92	67.65	65.23	60.31	54.08	45.51	44.02	43.62	42.90
C15	42.47	68.18	65.74	60.81	54.55	46.01	44.52	44.12	43.40
C2	119.74	136.25	137.43	133.41	131.00	118.56	117.37	120.28	118.93
C3	−5.35	23.86	19.83	14.36	5.71	0.06	−0.30	−5.08	−5.88
C7	139.52	154.53	155.12	151.36	150.01	136.84	134.75	140.23	138.47
C8	30.34	57.37	56.13	51.62	44.64	36.74	34.95	33.62	33.04
H10	29.31	29.67	28.51	26.73	28.50	29.43	28.62	29.40	28.85
H16	24.07	24.41	24.07	21.94	23.57	24.70	23.94	24.26	24.04
H17	23.89	24.14	23.71	21.55	23.16	24.34	23.64	23.90	23.74
H18	23.76	24.02	23.63	21.43	23.10	24.22	23.57	23.78	23.69
H19	23.85	24.15	23.76	21.56	23.08	24.34	23.70	23.90	23.82
H20	23.95	24.22	23.84	21.61	22.89	24.41	23.81	23.97	23.94
H22	30.77	31.20	29.86	27.28	30.14	30.21	30.42	30.61	30.35
H23	19.00	19.77	20.53	17.48	19.14	19.63	20.70	18.92	19.74
H5	29.70	30.05	28.80	26.32	28.92	29.20	29.52	29.59	29.48
H6	28.19	28.53	27.49	25.79	27.42	28.50	27.91	28.09	27.86
H9	27.46	27.76	26.76	25.07	26.60	27.75	26.83	27.72	27.04
N1	206.79	222.47	217.36	211.11	213.46	205.75	203.41	208.24	205.66
O21	104.80	134.59	126.05	115.69	111.98	95.74	93.92	105.35	103.36
O4	−103.51	−55.79	−58.80	−70.18	−83.96	−90.48	−91.26	−103.56	−104.35

See footnote of Table S1.

Table S14: L-Tyrosine, Cartesian coordinates and nuclear magnetic shielding constants.

Atm	x	y	z
N1	3.2459627	4.9055398	−3.0918196
C2	2.3906203	2.4605566	−2.0088965
C3	2.5022984	0.4077382	−4.0686922
O4	2.4866120	0.8618453	−6.3384342
H5	5.0181549	4.6545163	−3.8229592
H6	3.7347850	1.9634729	−0.5142348
C7	−0.3123947	2.7466288	−0.9866899
C8	−1.0921519	0.5719880	0.6951072
H9	−1.6625919	2.9402315	−2.5508320
H10	−0.4779336	4.4907371	0.1271677
C11	−2.4018045	−3.5233517	3.7536513
C12	−2.7440720	−1.2981520	−0.1989127
C13	−0.1196877	0.3675696	3.1516697
C14	−0.7731515	−1.6819015	4.6751919
C15	−3.3975966	−3.3485731	1.3284857
H16	−3.5253163	−1.1806953	−2.0993599
H17	1.1420274	1.8087835	3.9021247
H18	−0.0251607	−1.8367714	6.5815421
H19	−4.6743406	−4.7780285	0.5958621
O20	−2.9816491	−5.4951486	5.3053304
H21	−4.1911307	−6.5814909	4.4523258
O22	2.6522618	−1.9791805	−3.1694017
H23	2.5657265	−2.1072363	−1.3262002
H24	2.1671444	5.2581038	−4.6630054

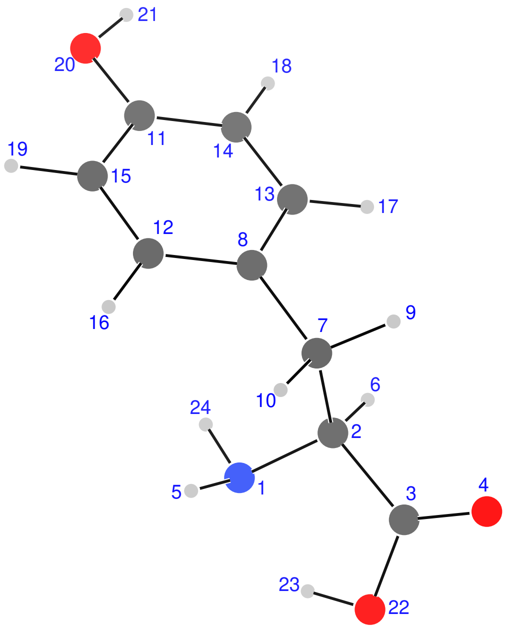

Atm	λ	GIAO	CSGT	DZ2	PZ2	GRRO $\bar{\alpha}$	GPRO $\bar{\beta}$	GRRO $\bar{\alpha}_{\mathrm{CE}}$	GPRO $\bar{\beta}_{\mathrm{CE}}$
C11	16.38	44.59	40.94	35.48	27.56	20.62	20.44	16.76	16.35
C12	38.68	64.39	62.18	57.35	51.07	42.63	41.18	40.75	40.02
C13	41.74	67.63	65.23	60.34	54.09	45.60	44.21	43.72	43.05
C14	59.49	83.49	79.28	74.14	68.68	59.23	58.35	57.36	57.17
C15	58.39	82.08	78.60	73.48	68.11	58.61	57.68	56.74	56.52
C2	119.19	135.75	137.00	132.96	130.48	118.11	117.02	119.83	118.58
C3	−5.34	23.89	19.88	14.39	5.69	0.09	−0.19	−5.05	−5.77
C7	140.15	155.04	155.73	151.92	150.59	137.41	135.46	140.80	139.17
C8	39.66	65.53	63.80	59.18	52.64	44.26	42.75	41.15	40.82
H10	29.43	29.80	28.66	26.85	28.63	29.55	28.88	29.52	29.09
H16	24.09	24.41	24.07	21.92	23.58	24.66	24.11	24.23	24.19
H17	24.02	24.30	23.91	21.72	23.29	24.47	23.98	24.04	24.07
H18	24.66	24.99	24.59	22.18	23.50	24.95	24.81	24.51	24.89
H19	24.32	24.53	24.11	21.79	23.51	24.57	24.42	24.14	24.50
H21	27.10	27.58	27.44	24.17	25.20	26.69	28.70	26.75	27.78
H23	19.03	19.81	20.58	17.52	19.18	19.67	20.80	18.96	19.84
H24	29.64	30.01	28.80	26.31	28.90	29.18	29.59	29.57	29.54
H5	30.81	31.25	29.93	27.34	30.19	30.26	30.56	30.67	30.49
H6	28.34	28.70	27.68	25.96	27.57	28.66	28.19	28.26	28.14
H9	27.56	27.89	26.94	25.22	26.76	27.90	27.11	27.87	27.31
N1	207.13	222.78	217.71	211.44	213.79	206.07	203.82	208.57	206.07
O20	215.39	237.23	221.30	210.69	213.49	190.83	189.82	216.44	214.25
O22	104.82	134.65	126.06	115.68	111.89	95.73	93.97	105.34	103.41
O4	−103.18	−55.50	−58.53	−69.92	−83.69	−90.22	−90.93	−103.30	−104.02

See footnote of Table S1.

Table S15: L-Tryptophan, Cartesian coordinates and nuclear magnetic shielding constants.

Atm	x	y	z
N1	−4.4891525	−0.3148267	−1.1272011
C2	−4.0213869	2.2258276	0.0545930
C3	−5.7114918	4.1643600	−1.2893019
O4	−5.5107857	6.4567030	−1.1431993
H5	−3.3579808	−0.4471120	−2.6977795
H6	−4.6730474	2.1249848	2.0188743
C7	−1.2489038	3.0764369	−0.0801893
C8	0.5157521	1.2845771	1.2244524
H9	−1.0532635	4.9493782	0.7919626
H10	−0.6750228	3.3211330	−2.0585099
C11	0.9255494	1.1278504	3.7981572
C12	2.0844049	−0.5927649	0.0640236
H13	0.1441008	2.1999904	5.3550972
N14	2.6454806	−0.7533641	4.2363192
C15	3.3692323	−1.8481708	1.9939222
H16	3.3018434	−1.2703510	5.9540278
C17	2.5267069	−1.3499314	−2.4533413
C18	5.0615248	−3.8298747	1.5373154
H19	6.0442572	−4.7775536	3.0675407
C20	5.4567597	−4.5495689	−0.9727013
H21	6.7646367	−6.0793506	−1.3909708
C22	4.2128962	−3.3283464	−2.9434463
H23	1.5895247	−0.4043562	−4.0142699
H24	4.5606970	−3.9088300	−4.8837272
O25	−7.5376207	2.9914484	−2.6675532
H26	−7.2321814	1.1559641	−2.4410635
H27	−3.6925295	−1.6242526	0.0669689

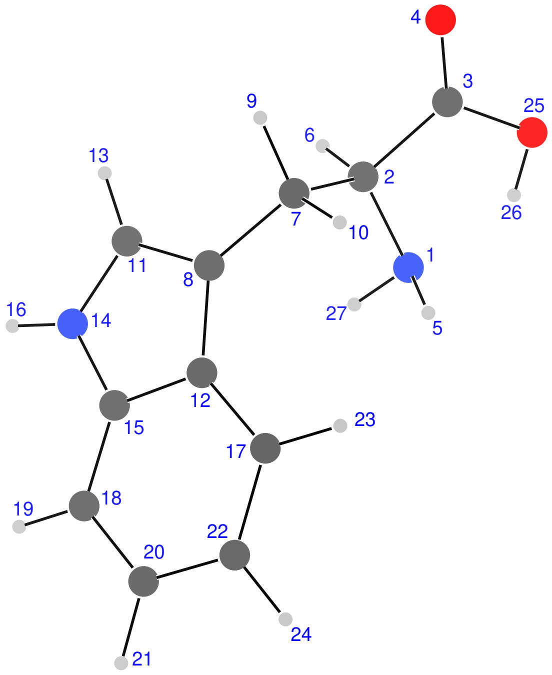

Atm	λ	GIAO	CSGT	DZ2	PZ2	GRRO $\bar{\alpha}$	GPRO $\bar{\beta}$	GRRO $\bar{\alpha}_{\mathrm{CE}}$	GPRO $\bar{\beta}_{\mathrm{CE}}$
C11	52.02	76.32	72.02	66.85	60.74	52.32	51.12	53.47	52.14
C12	46.46	71.27	67.84	62.66	56.10	47.76	46.23	44.65	44.26
C15	38.01	63.92	60.17	54.73	47.52	39.77	38.69	37.35	36.66
C17	53.95	78.29	75.76	70.76	64.82	56.00	54.52	54.12	53.36
C18	62.74	86.37	83.13	78.03	72.46	63.15	61.98	61.27	60.82
C2	116.23	132.72	134.13	130.09	127.60	115.26	114.02	116.98	115.57
C20	49.53	74.43	71.76	66.64	60.39	51.82	50.61	49.94	49.47
C22	52.07	76.64	73.93	68.83	62.74	54.00	52.72	52.11	51.58
C3	−5.63	23.54	19.44	13.98	5.35	−0.31	−0.76	−5.44	−6.34
C7	152.13	165.88	166.51	162.67	162.06	148.16	146.15	151.54	149.84
C8	58.94	82.91	80.25	75.38	69.61	60.44	59.00	57.34	57.01
H10	28.85	29.17	27.99	26.23	27.98	28.90	28.11	28.87	28.30
H13	24.07	24.30	24.01	21.79	23.20	24.51	24.28	24.04	23.87
H16	23.63	24.18	23.67	21.21	22.75	23.86	24.08	23.66	23.49
H19	23.95	24.25	23.85	21.57	23.08	24.35	23.90	23.91	24.01
H21	23.90	24.20	23.84	21.52	23.03	24.32	23.85	23.87	23.97
H23	23.72	24.01	23.60	21.35	22.82	24.10	23.44	23.66	23.52
H24	23.93	24.21	23.90	21.59	23.14	24.38	23.85	23.94	23.95
H26	18.89	19.69	20.39	17.36	18.99	19.51	20.51	18.80	19.56
H27	29.52	29.83	28.56	26.12	28.70	28.98	29.22	29.37	29.15
H5	30.53	30.92	29.56	27.00	29.84	29.91	30.03	30.31	29.95
H6	28.35	28.66	27.62	25.91	27.60	28.62	28.01	28.22	27.95
H9	27.52	27.83	26.90	25.16	26.81	27.84	27.02	27.80	27.21
N1	206.29	221.98	216.72	210.49	212.86	205.14	202.72	207.64	204.96
N14	116.79	143.02	137.03	129.92	126.72	123.40	121.45	115.50	113.89
O25	104.18	133.82	125.61	115.28	111.42	95.34	93.42	104.94	102.87
O4	−101.43	−54.14	−57.08	−68.43	−82.24	−88.73	−89.61	−101.81	−102.69

See footnote of Table S1.

Table S16: L-Aspartic acid, Cartesian coordinates and nuclear magnetic shielding constants.

Atm	x	y	z
N1	−2.4498780	−2.8396145	0.8494128
C2	−0.2589192	−1.3338688	−0.1397835
C3	1.9506365	−1.6638257	1.7262321
O4	3.7929664	−0.2877255	1.8855469
H5	−3.3301616	−1.8072307	2.2352606
H6	0.3250100	−2.1829708	−1.9351028
C7	−0.9382582	1.4554122	−0.3934945
C8	0.9654560	2.7823570	−2.0319585
H9	−0.9952765	2.3639370	1.4682372
H10	−2.7868057	1.7001386	−1.2949896
O11	2.0620277	1.8929829	−3.8521606
O12	1.2878266	5.2150467	−1.3661589
H13	2.6056446	5.8358442	−2.5122812
O14	1.5969784	−3.7290551	3.2134002
H15	−3.7675440	−2.9597788	−0.5649202
H16	−0.0597028	−4.4416486	2.7127600

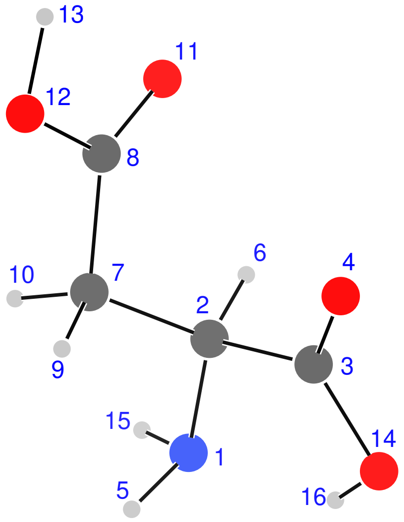

Atm	λ	GIAO	CSGT	DZ2	PZ2	GRRO $\bar{\alpha}$	GPRO $\bar{\beta}$	GRRO $\bar{\alpha}_{\mathrm{CE}}$	GPRO $\bar{\beta}_{\mathrm{CE}}$
C2	125.67	142.08	143.34	139.19	137.14	124.29	123.84	126.01	125.35
C3	−5.33	23.97	19.74	14.21	5.30	−0.07	0.09	−5.21	−5.52
C7	141.87	156.42	156.56	152.50	151.50	137.84	136.88	141.23	140.51
C8	−3.94	25.06	21.20	15.81	7.39	1.70	1.73	−3.45	−3.96
H10	29.27	29.66	28.60	26.67	28.55	29.40	29.35	29.37	29.52
H13	24.56	24.96	25.05	21.89	24.07	24.24	26.35	23.43	24.90
H15	29.61	30.02	28.91	26.19	28.96	29.11	29.99	29.52	29.91
H16	19.38	20.15	20.90	17.84	19.45	19.99	21.46	19.28	20.47
H5	30.23	30.66	29.48	26.74	29.68	29.69	30.48	30.10	30.38
H6	27.39	27.76	26.93	25.18	26.78	27.79	27.85	27.40	27.75
H9	28.69	29.06	28.06	26.05	27.91	28.72	28.94	28.68	29.10
N1	198.45	215.50	210.58	204.13	206.29	198.69	197.01	201.19	199.23
O11	−107.71	−58.28	−61.43	−72.71	−86.64	−93.04	−93.42	−106.12	−106.63
O12	110.84	138.68	130.90	120.54	117.21	100.67	99.46	110.23	108.71
O14	106.05	135.96	126.32	115.93	112.03	95.97	94.55	105.57	103.98
O4	−110.29	−63.44	−64.86	−76.21	−90.14	−96.47	−96.91	−109.55	−110.01

See footnote of Table S1.

Table S17: L-Glutamic acid, Cartesian coordinates and nuclear magnetic shielding constants.

Atm	x	y	z
N1	1.9566389	2.4175991	0.5686256
C2	1.6393857	−0.0166417	1.9474016
C3	3.6069917	−1.9099389	0.9430135
O4	4.0055303	−4.0066478	1.8153567
H5	0.2732395	3.3969919	0.6630519
H6	2.0913340	0.3236005	3.9405650
C7	−1.0378681	−1.1238938	1.7524702
C8	−2.0605287	−1.4439316	−0.9391811
H9	−2.3371442	0.0506416	2.8632802
H10	−1.0340817	−2.9933755	2.6521041
H11	−3.6161200	−2.8115829	−0.8884854
H12	−0.5976750	−2.1621088	−2.2124193
C13	−3.1261436	0.9712195	−2.0044302
O14	−3.2653673	3.0162230	−0.9469284
O15	−3.9862976	0.6675467	−4.3789432
H16	−4.6303010	2.3336587	−4.8673697
O17	4.8351818	−0.9670376	−1.1063082
H18	4.1007288	0.7334524	−1.3870024
H19	3.1824965	3.5242251	1.5851990

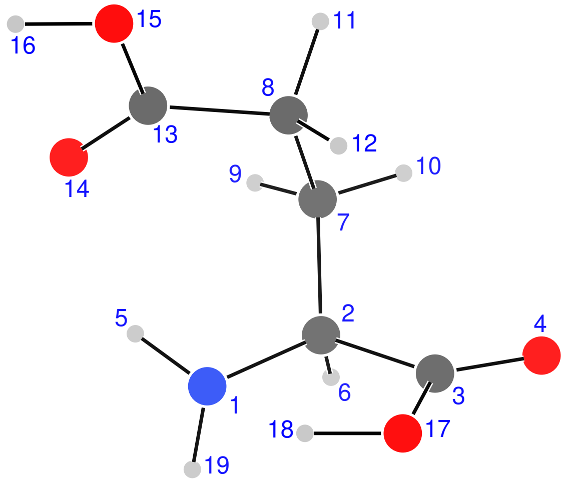

Atm	λ	GIAO	CSGT	DZ2	PZ2	GRRO $\bar{\alpha}$	GPRO $\bar{\beta}$	GRRO $\bar{\alpha}_{\mathrm{CE}}$	GPRO $\bar{\beta}_{\mathrm{CE}}$
C13	−10.80	18.84	15.04	9.85	1.18	−4.23	−4.37	−9.37	−10.00
C2	125.27	141.29	142.68	138.57	136.56	123.73	123.25	125.45	124.81
C3	−6.37	23.03	18.87	13.29	4.52	−0.98	−0.66	−6.12	−6.24
C7	156.23	170.00	171.31	167.44	167.36	152.90	151.90	156.30	155.64
C8	154.26	168.26	168.24	164.29	164.17	149.69	148.73	153.08	152.44
H10	29.33	29.65	28.51	26.56	28.52	29.38	29.47	29.35	29.71
H11	29.07	29.34	28.29	26.35	28.27	29.14	29.17	29.10	29.39
H12	29.07	29.31	28.03	26.14	28.12	28.90	29.01	28.86	29.20
H16	24.63	24.99	25.06	21.98	24.15	24.33	26.41	23.52	24.96
H18	18.56	19.32	20.08	17.10	18.72	19.22	20.64	18.52	19.72
H19	30.08	30.51	29.40	26.76	29.44	29.67	30.37	30.08	30.31
H5	27.60	28.06	27.41	24.60	26.99	27.30	28.08	27.67	28.01
H6	27.87	28.33	27.35	25.57	27.16	28.31	28.35	27.90	28.30
H9	29.18	29.49	28.29	26.34	28.29	29.14	29.15	29.11	29.39
N1	210.93	226.55	222.72	216.36	218.91	210.94	209.02	213.44	211.26
O14	−100.82	−51.86	−54.43	−65.29	−79.19	−85.63	−86.54	−98.70	−99.67
O15	113.48	141.31	134.11	123.82	120.78	103.95	102.71	113.52	111.97
O17	99.10	129.61	122.26	111.95	107.82	92.02	90.53	101.63	99.99
O4	−102.55	−55.85	−58.58	−70.16	−84.04	−90.44	−90.34	−103.52	−103.45

See footnote of Table S1.

Table S18: L-Histidine, Cartesian coordinates and nuclear magnetic shielding constants.

Atm	x	y	z
N1	−2.0702398	0.9506183	−3.4912704
C2	0.7343867	0.7804783	−3.0651375
C3	1.5240674	−2.0093424	−3.2140111
O4	3.6095108	−2.8424130	−2.6817300
H5	−2.8875623	0.6566425	−1.7294638
H6	1.6506651	1.7317564	−4.6609510
C7	1.6206588	1.9676455	−0.5706659
C8	0.4320770	0.8061220	1.7060468
H9	1.1764357	3.9930215	−0.5790218
H10	3.6833548	1.8162976	−0.4217781
C11	1.5486099	−0.4851942	3.6703411
N12	−2.1505991	0.8899355	2.0322628
H13	3.4925148	−0.9718267	4.0713202
H14	−0.2565158	−2.2010136	6.8305523
C15	−2.5847971	−0.3220924	4.1552338
N16	−0.3979128	−1.1956756	5.2122068
H17	−4.4158489	−0.6325549	5.0108217
O18	−0.3945768	−3.4664087	−4.1102827
H19	−1.8264623	−2.2814583	−4.3630340
H20	−2.4877661	2.8154625	−3.8014392

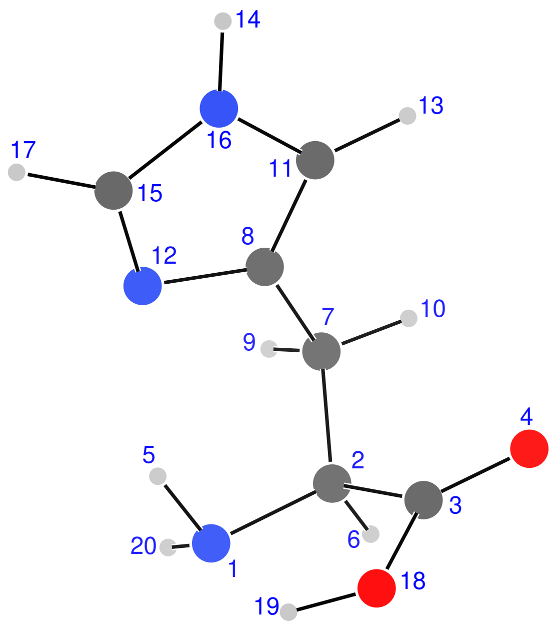

Atm	λ	GIAO	CSGT	DZ2	PZ2	GRRO $\bar{\alpha}$	GPRO $\bar{\beta}$	GRRO $\bar{\alpha}_{\rm CE}$	GPRO $\bar{\beta}_{\rm CE}$
C11	60.77	84.08	79.36	74.03	68.49	59.24	58.97	60.39	59.93
C15	37.72	63.63	59.17	53.85	47.18	39.40	38.81	38.20	37.28
C2	122.08	138.24	139.70	135.58	133.36	120.70	120.04	122.42	121.57
C3	−6.42	23.02	18.88	13.36	4.54	−0.86	−0.75	−6.00	−6.35
C7	152.32	166.29	166.95	163.02	162.49	148.47	147.08	151.85	150.75
C8	31.90	58.68	55.68	50.61	43.57	35.62	35.05	33.22	32.95
H10	28.09	28.42	27.46	25.63	27.36	28.31	28.19	28.28	28.39
H13	24.41	24.63	24.54	22.29	23.68	24.98	25.34	24.52	24.89
H14	22.73	23.26	22.93	20.52	21.91	23.14	23.91	22.93	23.26
H17	23.93	24.19	24.16	21.89	23.44	24.59	24.84	23.97	24.14
H19	19.07	19.86	20.68	17.54	19.12	19.71	21.19	19.00	20.20
H20	30.08	30.38	29.13	26.41	29.19	29.33	30.02	29.73	29.94
H5	25.82	26.33	25.25	22.48	24.87	25.20	25.68	25.57	25.57
H6	28.00	28.36	27.34	25.59	27.26	28.31	28.17	27.90	28.09
H9	28.94	29.25	27.99	26.15	27.92	28.90	28.63	28.87	28.83
N1	206.56	222.02	216.81	210.40	212.85	205.06	203.07	207.55	205.29
N12	−45.70	−2.46	−4.45	−11.48	−22.32	−17.90	−19.82	−45.44	−45.83
N16	80.72	110.60	104.97	97.81	93.13	91.36	90.10	83.44	82.41
O18	98.71	128.33	120.64	110.23	106.03	90.35	88.92	99.96	98.34
O4	−97.11	−50.44	−52.30	−63.75	−77.15	−84.04	−84.32	−97.11	−97.43

See footnote of Table S1.

Table S19: L-Lysine, Cartesian coordinates and nuclear magnetic shielding constants.

Atm	x	y	z
N1	−0.1984568	0.0137897	−4.9239399
C2	0.9486529	2.0204114	−3.2996674
C3	3.3197510	3.0347285	−4.6536940
O4	4.9426935	4.3718362	−3.7074855
H5	0.6825831	−1.6649647	−4.5136148
H6	−0.3889880	3.5978450	−3.1895648
C7	1.6220667	1.0493082	−0.6547074
H8	2.5735933	2.5630737	0.3972884
H9	2.9969671	−0.4983822	−0.7973648
C10	−0.6848618	0.1660553	0.8540266
H11	−1.5851401	−1.4253393	−0.1164605
H12	−2.0773247	1.6981399	0.9488221
C13	0.0579788	−0.6293041	3.5380501
H14	1.4517641	−2.1624042	3.4529688
C15	−2.2066464	−1.4541697	5.1336298
H16	0.9824362	0.9634842	4.4899722
H17	−3.5963653	0.0766668	5.2320163
H18	−1.5781908	−1.8468244	7.0666361
N19	−3.4111366	−3.7087568	4.1016008
H20	−2.1643176	−5.1776393	4.1434016
H21	−4.8720859	−4.2250412	5.2481869
O22	3.3665798	2.2777089	−7.1098408
H23	1.8331235	1.2249666	−7.3306529
H24	−2.0146763	−0.2651884	−4.3096070

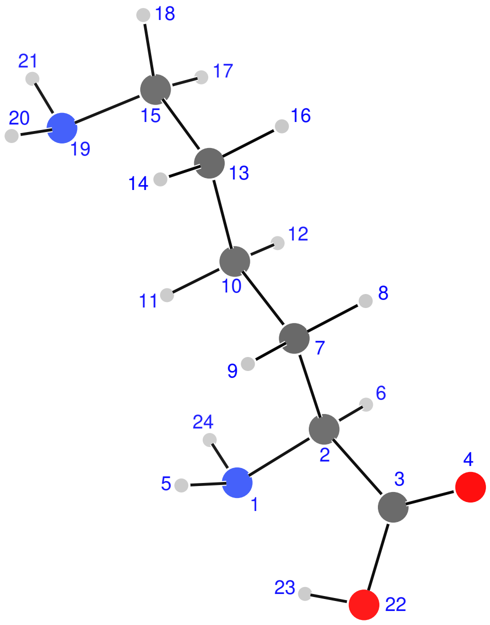

Atm	λ	GIAO	CSGT	DZ2	PZ2	GRRO $\bar{\alpha}$	GPRO $\bar{\beta}$	GRRO $\bar{\alpha}_{\mathrm{CE}}$	GPRO $\bar{\beta}_{\mathrm{CE}}$
C10	154.54	169.02	170.88	167.11	167.02	152.76	151.43	156.17	155.29
C13	147.33	162.06	164.58	160.70	160.26	146.33	145.20	149.75	149.05
C15	138.58	154.16	155.30	151.05	149.96	136.84	136.27	140.50	139.29
C2	121.16	137.68	139.32	135.31	133.06	120.53	119.74	122.26	121.33
C3	−5.86	23.40	19.23	13.73	4.94	−0.52	−0.55	−5.66	−6.12
C7	143.83	158.93	160.72	156.91	156.17	142.51	141.16	145.91	144.96
H11	29.38	29.58	27.96	26.10	28.04	29.10	28.70	29.07	29.00
H12	30.79	30.96	29.16	27.17	29.32	30.19	29.94	30.17	30.26
H14	30.42	30.61	28.77	26.67	28.81	29.73	29.62	29.71	29.96
H16	30.00	30.28	28.62	26.56	28.69	29.60	29.37	29.58	29.70
H17	29.21	29.38	27.73	25.51	27.51	28.65	28.78	28.67	28.79
H18	28.39	28.72	27.00	24.75	26.62	27.94	28.16	27.96	28.17
H20	31.39	31.67	29.96	26.89	30.18	30.13	31.47	30.59	31.46
H21	30.57	30.93	29.36	26.33	29.42	29.52	30.82	29.97	30.80
H23	18.83	19.61	20.36	17.32	18.90	19.49	20.78	18.79	19.83
H24	29.06	29.47	28.31	25.74	28.35	28.66	29.18	29.07	29.14
H5	30.81	31.19	29.82	27.19	30.11	30.17	30.73	30.59	30.68
H6	28.54	28.86	27.70	26.01	27.71	28.76	28.49	28.36	28.46
H8	29.12	29.41	28.11	26.18	28.16	29.07	28.81	29.04	29.09
H9	30.64	30.89	29.32	27.35	29.43	30.27	30.06	30.24	30.35
N1	205.54	221.69	216.81	210.50	212.87	205.10	203.10	207.60	205.37
N19	222.27	236.96	228.56	221.69	225.21	216.30	215.32	218.84	217.68
O22	106.68	136.42	127.96	117.59	113.89	97.66	96.06	107.27	105.52
O4	−101.41	−54.11	−57.02	−68.46	−82.04	−88.74	−89.15	−101.82	−102.23

See footnote of Table S1.

Table S20: L-Arginine, Cartesian coordinates and nuclear magnetic shielding constants.

Atm	x	y	z
N1	3.7540059	−4.0729172	−0.4752391
C2	2.1710338	−1.7289992	−0.2982797
C3	3.0594938	0.0999347	−2.3845338
O4	1.9107723	1.9713798	−3.1037265
H5	3.4070717	−5.1464738	1.0985190
H6	0.2190913	−2.2915942	−0.6720261
C7	2.4934185	−0.5692656	2.3395912
H8	4.5048256	−0.1830525	2.6797372
H9	1.9243664	−1.9692014	3.7623653
C10	1.0390576	1.8909160	2.8753855
H11	1.8218226	3.4382937	1.7412757
H12	1.4001054	2.3950357	4.8548913
C13	−1.8238634	1.7163794	2.5121170
H14	−2.5247126	−0.0137452	3.4087320
N15	−2.4560572	1.7736212	−0.1360106
H16	−2.7336654	3.3239953	3.4464649
C17	−4.6122478	0.9721524	−1.2203313
H18	−1.1872092	2.6408235	−1.3009866
N19	−6.5903041	0.1198759	−0.1129178
N20	−4.5285641	1.0848918	−3.7440158
H21	−6.3329888	0.1944923	1.7934217
H22	−3.0163746	1.7238722	−4.7118077
H23	−6.0858149	0.5369878	−4.7019594
O24	5.3446621	−0.5992197	−3.3221818
H25	5.8083098	−2.1638333	−2.4026320
H26	3.0337652	−5.1443496	−1.9258528

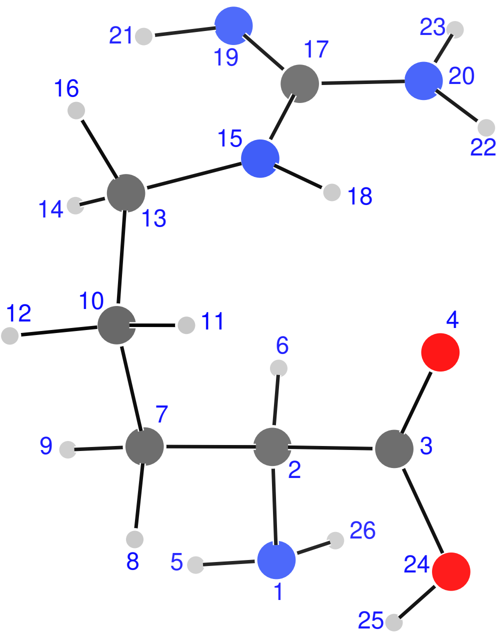

Atm	λ	GIAO	CSGT	DZ2	PZ2	GRRO $\bar{\alpha}$	GPRO $\bar{\beta}$	GRRO $\bar{\alpha}_{\rm CE}$	GPRO $\bar{\beta}_{\rm CE}$
C10	156.28	170.59	172.01	168.20	168.21	153.76	152.44	157.17	156.25
C13	142.42	157.03	157.61	153.41	152.57	139.33	138.10	142.94	141.06
C17	21.82	50.18	45.61	39.72	33.20	25.41	25.60	22.47	21.74
C2	126.03	142.54	143.68	139.86	138.29	125.16	124.29	126.88	125.90
C3	−9.20	20.30	16.45	11.33	2.70	−2.73	−3.19	−7.86	−8.70
C7	146.78	161.64	163.85	160.09	159.73	145.61	144.39	149.02	148.20
H11	29.74	29.99	28.54	26.61	28.74	29.47	29.19	29.44	29.47
H12	29.94	30.27	28.84	26.86	28.95	29.81	29.54	29.78	29.85
H14	28.81	29.20	27.85	25.68	27.59	28.56	28.53	28.57	28.52
H16	28.42	28.71	27.61	25.54	27.40	28.50	28.36	28.51	28.35
H18	25.34	26.03	25.80	22.85	25.40	25.49	26.10	25.11	25.78
H21	28.11	28.11	27.97	24.41	26.96	27.35	28.47	27.59	28.34
H22	27.88	28.36	27.60	24.30	28.83	27.26	28.57	27.71	28.17
H23	27.53	27.94	27.19	23.85	28.09	26.82	28.22	27.28	27.80
H25	18.98	19.76	20.26	17.43	19.13	19.57	20.78	18.88	19.91
H26	29.79	30.19	28.88	26.35	29.59	29.32	29.99	29.74	29.94
H5	30.04	30.49	29.19	26.65	29.73	29.56	30.24	29.97	30.21
H6	27.39	27.75	26.44	25.04	27.09	27.76	27.45	27.38	27.43
H8	29.79	30.06	28.52	26.65	28.87	29.52	29.47	29.50	29.78
H9	30.10	30.43	28.96	27.01	29.26	29.93	29.80	29.90	30.10
N1	202.85	219.12	214.44	208.23	210.99	202.90	200.89	205.41	203.17
N15	171.01	190.73	186.16	179.23	179.46	172.73	171.20	171.84	170.47
N19	110.50	136.60	125.23	117.21	114.90	111.33	110.53	111.41	109.97
N20	191.53	209.94	203.33	195.77	200.69	189.75	189.06	193.02	190.56
O24	106.66	136.26	127.46	117.35	113.35	97.46	95.65	107.07	105.15
O4	−101.57	−53.67	−57.05	−67.87	−81.54	−88.09	−89.42	−101.15	−102.41

See footnote of Table S1.

Table S21: GRRO and GPRO constants for Hydrogen atom in different chemical environments.

Chemical environment	α	β
'H '	'-0.369649680 '	'-0.060982769 '
'H-CCHH primary '	'-0.416157286 '	'-0.072088992 '
'H-CCCH secondary '	'-0.367274738 '	'-0.067760585 '
'H-CCCC tertiary '	'-0.394331727 '	'-0.078079156 '
'H-CNHH meth-amine '	'-0.369649680 '	'-0.060982769 '
'H-CNCH prim-amine '	'-0.370595238 '	'-0.060989094 '
'H-CNCC seco-amine '	'-0.328105057 '	'-0.060955645 '
'H-COHH meth-oxyx '	'-0.369649680 '	'-0.060982769 '
'H-COCH prim-oxyx '	'-0.353567509 '	'-0.053063001 '
'H-COCC seco-oxyx '	'-0.319895013 '	'-0.057783116 '
'H-CSHH meth-tio '	'-0.466090613 '	'-0.070804765 '
'H-CSCH prim-tio '	'-0.443710844 '	'-0.072146504 '
'H-CSCC seco-tio '	'-0.369649680 '	'-0.060982769 '
'H-CCC aroma '	'-0.327723473 '	'-0.063628860 '
'H-CNC aroma '	'-0.323139326 '	'-0.053327115 '
'H-CNN aroma '	'-0.308043912 '	'-0.048161902 '
'H-N=CNN guanidine '	'-0.390741325 '	'-0.059235082 '
'H-N=C= imine '	'-0.369649680 '	'-0.060982769 '
'H-NCH amine1 '	'-0.408818073 '	'-0.060627204 '
'H-NCC amine2 '	'-0.379096958 '	'-0.060224098 '
'HH=N-CNN guanidine'	'-0.411221056 '	'-0.054938314 '
'HC=N-CNN guanidine'	'-0.328148488 '	'-0.052706662 '
'H-N-CC aroma '	'-0.349789111 '	'-0.050550269 '
'H-N-CO amide '	'-0.352272388 '	'-0.050219161 '
'H-O carboxyl '	'-0.282310812 '	'-0.037193251 '
'H-O phenyl '	'-0.376234004 '	'-0.045847443 '
'H-O alcoh1 '	'-0.423755310 '	'-0.048715119 '
'H-O alcoh2 '	'-0.413499563 '	'-0.053530331 '
'H-O alcoh3 '	'-0.369649680 '	'-0.060982769 '
'H-S tio1 '	'-0.539450172 '	'-0.070385781 '

Table S22: GRRO and GPRO constants for Oxygen atom in different chemical environments.

Chemical environment	α	β
'O '	'-0.004147947 '	'-0.073165026 '
'O=C(-)-O carboxyl '	'-0.003931427 '	'-0.069331372 '
'O=C(-)-N amide '	'-0.003946791 '	'-0.069468897 '
'O=C= carbonyl '	'-0.004147947 '	'-0.073165026 '
'OH-C=O carboxyl '	'-0.004310542 '	'-0.076093182 '
'OH-C(-C)-C phenyl '	'-0.004574182 '	'-0.080538984 '
'OH-CHHC alcohol1 '	'-0.004790300 '	'-0.083998610 '
'OH-CHCC alcohol2 '	'-0.004668036 '	'-0.082270706 '
'OH-CCCC alcohol3 '	'-0.004147947 '	'-0.073165026 '

Table S23: GRRO and GPRO constants for Carbon atom in different chemical environments.

Chemical environment	α	β
'C '	'-0.006799643 '	'-0.067410214 '
'O-(C=O)-H carboxyl'	'-0.006799643 '	'-0.067410214 '
'O-(C=O)-C carboxyl'	'-0.006583671 '	'-0.065101162 '
'N-(C=O)-H amide '	'-0.006800000 '	'-0.067410214 '
'N-(C=O)-C amide '	'-0.006555008 '	'-0.064763950 '
'H-(C=N)-H imine '	'-0.006799643 '	'-0.067410214 '
'C-(C=N)-H imine '	'-0.006799643 '	'-0.067410214 '
'C-(C=N)-C imine '	'-0.006799643 '	'-0.067410214 '
'N-(C=N)-Nguanidine'	'-0.006673626 '	'-0.065814314 '
'C-(C-C)-H aroma '	'-0.006723359 '	'-0.066919029 '
'C-(C-C)-C aroma '	'-0.006669824 '	'-0.066593676 '
'C-(C-C)-O aroma '	'-0.006638461 '	'-0.065698160 '
'N-(C-C)-H aroma '	'-0.006850726 '	'-0.067928989 '
'N-(C-C)-C aroma '	'-0.006699333 '	'-0.066583243 '
'N-(C-N)-H aroma '	'-0.006752168 '	'-0.066912762 '
'NC=C=HH prim-amine'	'-0.006952291 '	'-0.068678223 '
'NC=C=CH seco-amine'	'-0.006872600 '	'-0.068103046 '
'NC=C=CC tert-amine'	'-0.006799643 '	'-0.067410214 '
'CH=C=HH primary '	'-0.007023824 '	'-0.069591144 '
'CC=C=HH secondary '	'-0.006943064 '	'-0.068978847 '
'CC=C=CH tertiary '	'-0.006791687 '	'-0.067405179 '
'OC=C=HH prim-oxy '	'-0.006907549 '	'-0.067955147 '
'OC=C=CH seco-oxy '	'-0.006823971 '	'-0.067507287 '
'OC=C=CC tert-oxy '	'-0.006799643 '	'-0.067410214 '
'SH=C=HH meth-tio '	'-0.007022306 '	'-0.068867865 '
'SC=C=HH prim-tio '	'-0.006897192 '	'-0.067922488 '

Table S24: GRRO and GPRO constants for Nitrogen atom in different chemical environments.

Chemical environment	α	β
'N '	'-0.005451939 '	'-0.073659463 '
'C-N-C aroma '	'-0.004752700 '	'-0.064622842 '
'NNC=N-H guanidine '	'-0.005452696 '	'-0.073498962 '
'=C=N-H imine '	'-0.005451939 '	'-0.073659463 '
'C-(N-H)-H amine1 '	'-0.005513613 '	'-0.074486497 '
'C-(N-C)-H amine2 '	'-0.005415746 '	'-0.073492435 '
'C-(N-C)-C amine3 '	'-0.005451939 '	'-0.073659463 '
'OC-N(-H)-H amide1 '	'-0.005375068 '	'-0.072455056 '
'NNC-N(-H)-H guanid'	'-0.005532528 '	'-0.074285801 '
'OC-N(-C)-H amide2 '	'-0.005451939 '	'-0.073659463 '
'NNC-C(-C)-H guanid'	'-0.005430044 '	'-0.073439000 '
'C-(N-C)-H aroma '	'-0.005250065 '	'-0.071072672 '

Table S25: GRRO and GPRO constants for Sulfur atom in different chemical environments.

Chemical environment	α	β
'S '	'-0.001697414 '	'-0.111740479 '
'C-S-H tio1 '	'-0.001717834 '	'-0.112955334 '
'C-S-C tio2 '	'-0.001676995 '	'-0.110525624 '

Table S26: BHandHLYP/6-31+G(d,p) mean absolute deviation in ppm for ^{13}C.

AA	GIAO	CSGT	DZ2	PZ2	GRRO $\bar{\alpha}$	GPRO $\bar{\beta}$	GRRO $\bar{\alpha}_{\text{CE}}$	GPRO $\bar{\beta}_{\text{CE}}$
Gly	22.28	20.07	14.84	9.76	4.00	4.47	0.40	0.21
Ala	19.80	19.14	14.48	11.29	3.42	3.89	0.73	0.62
Val	17.56	17.88	13.68	11.87	2.92	3.55	0.91	0.43
Leu	17.13	17.99	13.86	12.29	2.48	3.38	1.35	0.58
Ile	16.97	17.49	13.39	12.09	3.17	3.79	0.94	0.68
Pro	18.07	17.56	13.34	10.73	3.13	4.10	0.34	0.66
Ser	20.60	19.74	14.92	10.95	2.94	3.13	0.42	0.43
Thr	19.05	18.94	14.47	11.26	2.62	3.14	0.78	0.20
Asn	22.43	20.90	16.14	10.82	4.37	4.80	0.35	0.71
Gln	20.75	20.24	15.69	11.64	3.88	4.38	0.87	0.56
Cys	20.37	20.88	15.42	11.68	2.63	2.51	0.55	0.60
Met	18.00	19.24	14.16	12.52	2.40	2.54	1.40	0.89
Phe	23.99	22.17	17.48	11.89	3.62	2.89	1.37	0.96
Tyr	23.78	21.59	16.77	11.18	2.93	2.88	1.26	1.12
Trp	23.26	20.77	15.83	10.27	1.98	1.64	0.83	1.12
Asp	22.32	20.64	15.86	10.77	4.08	4.48	0.40	0.47
Glu	20.57	19.51	14.97	11.04	4.28	4.80	0.62	0.76
His	22.60	20.23	15.35	10.21	2.96	3.17	0.57	0.75
Lys	17.61	18.41	14.20	11.97	1.97	2.83	1.56	0.79
Arg	19.69	19.18	14.74	11.76	2.95	3.68	1.08	0.59
min	16.97	17.49	13.34	9.76	1.97	1.64	0.34	0.20
max	23.99	22.17	17.48	12.52	4.37	4.80	1.56	1.12
median	20.47	19.63	14.88	11.28	2.95	3.47	0.80	0.64

Table S27: BHandHLYP/6-31+G(d,p) mean absolute deviation in ppm for ^{1}H.

AA	GIAO	CSGT	DZ2	PZ2	GRRO $\bar{\alpha}$	GPRO $\bar{\beta}$	GRRO $\bar{\alpha}_{\text{CE}}$	GPRO $\bar{\beta}_{\text{CE}}$
Gly	0.47	0.97	3.14	0.81	0.49	1.55	0.24	1.26
Ala	0.39	1.21	3.29	0.86	0.56	0.58	0.22	0.58
Val	0.32	1.33	3.35	0.97	0.55	0.64	0.11	0.15
Leu	0.31	1.39	3.38	1.03	0.56	0.80	0.18	0.31
Ile	0.31	1.37	3.36	0.96	0.53	0.67	0.16	0.18
Pro	0.33	0.99	2.73	0.91	0.20	0.39	0.13	0.25
Ser	0.42	0.78	3.02	0.59	0.52	1.03	0.24	0.56
Thr	0.38	0.99	3.10	0.80	0.58	0.59	0.16	0.33
Asn	0.47	0.79	2.67	0.58	0.41	0.63	0.22	0.40
Gln	0.42	0.86	2.80	0.67	0.33	0.51	0.20	0.37
Cys	0.42	0.89	3.83	1.39	1.10	0.57	0.43	0.57
Met	0.41	1.15	3.63	1.24	0.85	0.67	0.31	0.34
Phe	0.36	0.55	2.47	0.70	0.45	0.43	0.10	0.26
Tyr	0.39	0.55	2.55	0.82	0.40	0.47	0.15	0.30
Trp	0.37	0.50	2.50	0.76	0.40	0.43	0.09	0.30
Asp	0.45	0.75	2.66	0.55	0.36	0.76	0.22	0.41
Glu	0.40	0.76	2.66	0.67	0.27	0.61	0.18	0.40
His	0.40	0.66	2.51	0.71	0.47	0.66	0.15	0.36
Lys	0.32	1.29	3.38	1.15	0.54	0.50	0.38	0.30
Arg	0.39	0.84	3.05	0.69	0.38	0.48	0.19	0.23
min	0.31	0.50	2.47	0.55	0.20	0.39	0.09	0.15
max	0.47	1.39	3.83	1.39	1.10	1.55	0.43	1.26
median	0.39	0.88	3.03	0.80	0.48	0.60	0.19	0.34

Table S28: BHandHLYP/6-31+G(d,p) mean absolute deviation in ppm for ^{15}N.

AA	GIAO	CSGT	DZ2	PZ2	GRRO $\bar{\alpha}$	GPRO $\bar{\beta}$	GRRO $\bar{\alpha}_{CE}$	GPRO $\bar{\beta}_{CE}$
Gly	14.77	8.59	1.75	5.43	3.61	3.84	1.09	1.63
Ala	17.25	12.66	6.14	8.35	0.75	0.54	3.28	1.78
Val	16.40	10.97	4.68	6.98	0.66	2.71	1.86	0.36
Leu	15.86	10.79	4.51	6.97	0.89	3.05	1.62	0.76
Ile	16.17	10.65	4.42	6.82	0.91	3.06	1.61	0.72
Pro	17.74	14.30	8.24	8.63	2.25	0.05	0.82	0.44
Ser	14.26	8.27	1.71	5.49	3.61	5.02	1.10	2.84
Thr	13.60	7.78	1.37	5.00	3.95	5.85	1.44	3.56
Asn	19.94	14.81	7.99	8.48	2.27	1.86	2.00	1.17
Gln	19.59	14.48	7.79	8.52	2.39	2.70	1.86	0.33
Cys	15.89	10.84	4.14	6.49	1.23	2.49	1.31	0.18
Met	16.90	12.86	6.42	8.54	1.04	0.74	3.55	1.58
Phe	15.67	10.57	4.32	6.66	1.05	3.39	1.45	1.13
Tyr	15.66	10.58	4.31	6.66	1.06	3.30	1.44	1.06
Trp	20.96	15.33	8.66	8.25	3.88	4.12	1.32	2.12
Asp	17.04	12.12	5.68	7.84	0.24	1.44	2.74	0.78
Glu	15.62	11.79	5.43	7.98	0.01	1.91	2.51	0.33
His	29.52	25.25	18.38	14.03	13.32	12.92	1.32	1.03
Lys	15.42	8.78	2.77	5.14	3.20	4.69	2.74	2.38
Arg	20.12	13.32	6.14	7.54	1.09	1.16	1.45	0.59
min	13.60	7.78	1.37	5.00	0.01	0.05	0.82	0.18
max	29.52	25.25	18.38	14.03	13.32	12.92	3.55	3.56
median	16.28	11.38	5.05	7.26	1.16	2.88	1.53	1.04

Table S29: BHandHLYP/6-31+G(d,p) mean absolute deviation in ppm for ^{17}O.

AA	GIAO	CSGT	DZ2	PZ2	GRRO $\bar{\alpha}$	GPRO $\bar{\beta}$	GRRO $\bar{\alpha}_{CE}$	GPRO $\bar{\beta}_{CE}$
Gly	39.39	34.22	22.86	13.87	12.06	12.87	0.95	1.59
Ala	38.66	33.09	22.00	13.46	11.21	11.91	0.14	0.65
Val	39.11	33.38	22.55	13.06	11.49	12.11	0.78	0.85
Leu	38.91	32.91	22.01	13.33	10.92	11.52	0.42	0.94
Ile	39.03	33.06	22.30	13.03	11.09	11.71	0.54	0.56
Pro	37.99	32.71	21.72	12.91	9.85	10.69	1.49	0.91
Ser	29.05	24.82	20.98	12.65	19.93	20.53	0.90	0.98
Thr	30.42	22.43	18.52	11.16	17.19	18.09	0.84	0.50
Asn	40.62	36.97	25.94	15.79	11.00	11.24	0.64	0.50
Gln	41.18	36.72	25.72	15.70	11.66	11.97	0.33	0.64
Cys	38.95	33.67	22.46	13.66	10.78	11.43	0.61	0.17
Met	38.40	33.29	22.29	13.25	10.40	11.04	0.94	0.23
Phe	38.75	32.98	22.11	13.36	11.04	11.57	0.30	1.14
Tyr	33.11	23.93	16.27	9.48	15.54	16.22	0.56	1.13
Trp	38.46	32.89	22.04	13.21	10.77	11.29	0.57	1.29
Asp	38.51	33.01	22.16	13.39	12.18	12.63	0.85	1.39
Glu	38.50	33.54	22.78	14.04	10.98	11.46	1.42	1.11
His	38.15	33.37	22.44	13.64	10.71	11.29	0.63	0.34
Lys	38.52	32.84	21.93	13.29	10.84	11.44	0.50	0.99
Arg	38.75	32.66	22.20	13.36	11.34	11.58	0.42	1.17
min	29.05	22.43	16.27	9.48	9.85	10.69	0.14	0.17
max	41.18	36.97	25.94	15.79	19.93	20.53	1.49	1.59
median	38.59	33.03	22.18	13.35	11.07	11.57	0.62	0.93

Table S30: Glutathione, Cartesian coordinates.

Atm	x	y	z
C1	4.5133305	−4.3944288	−1.5678933
C2	1.6865670	−4.7229861	−1.0325878
C3	0.0732505	−2.5360252	−1.9483161
O4	0.7693994	−0.9904860	−3.5332504
N5	−2.3012099	−2.4990460	−0.9051214
C6	−4.2520347	−0.7062790	−1.6864916
C7	−6.9247155	−1.6592660	−1.0702177
S8	−7.3027757	−2.8645536	2.1484433
C9	−3.7527669	1.9047563	−0.4932648
O10	−4.6998921	2.6150033	1.5033273
N11	−2.0259079	3.3440346	−1.7995317
C12	−1.1747824	5.8007255	−0.8979525
C13	1.2383985	5.5123086	0.4458208
O14	3.4591812	5.5791481	−0.7482063
O15	1.2754097	4.7869467	2.8695155
C16	6.0165485	−2.9364859	0.4560478
C17	5.2081342	−0.2422150	0.6631041
O18	3.6235239	0.5227885	2.4774875
O19	6.0708540	1.4598333	−0.9961345
N20	5.9746295	−4.3682190	2.8812680
H21	4.7982423	−3.4902745	−3.4118639
H22	5.3472299	−6.2888016	−1.7341666
H23	1.3186949	−5.0869576	0.9698586
H24	1.0151771	−6.3872485	−2.0735045
H25	−2.6962446	−3.6304931	0.5940773
H26	−4.1181656	−0.4777781	−3.7446936
H27	−7.3945880	−3.2276225	−2.3369845
H28	−8.3242516	−0.1656356	−1.3746833
H29	−6.7680073	−0.6640027	3.3096283
H30	−0.9681041	2.4822807	−3.1662732
H31	−2.5946456	6.6323886	0.3574222
H32	−0.9813302	7.0313966	−2.5516189
H33	2.1585341	3.1428068	2.9294886
H34	7.9990738	−2.9247642	−0.1595553
H35	5.1024693	3.0428765	−0.8094267
H36	4.3293244	−3.8154957	3.7616431
H37	7.3090320	−3.5837016	4.0378490

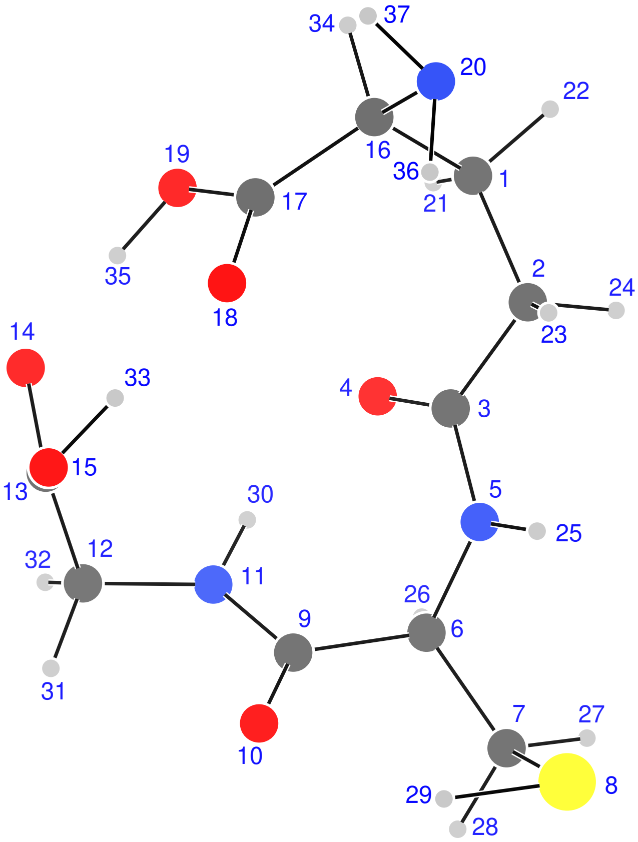

Table S31: Glutathione, nuclear magnetic shielding constants.

Atm	λ	GIAO	CSGT	DZ2	PZ2	GRRO $\bar{\alpha}$	GPRO $\bar{\beta}$	GRRO $\bar{\alpha}_{\mathrm{CE}}$	GPRO $\bar{\beta}_{\mathrm{CE}}$
C1	153.57	167.42	168.79	165.10	164.68	150.64	148.94	154.05	152.73
C12	141.73	155.89	153.89	149.67	148.29	135.27	133.85	138.88	136.79
C13	−50.90	−18.31	−21.28	−26.33	−37.84	−40.64	−41.13	−45.79	−46.66
C16	129.18	144.97	146.16	142.19	140.22	127.44	126.24	129.17	127.87
C17	−48.08	−15.27	−18.56	−23.48	−34.23	−37.58	−38.29	−42.73	−43.82
C2	153.49	167.23	167.19	163.46	163.06	149.02	147.21	152.43	151.01
C3	−4.89	24.39	21.96	17.11	8.56	3.10	1.62	−2.67	−4.54
C6	131.27	146.16	146.82	142.43	140.62	127.87	126.59	129.62	128.26
C7	152.46	166.54	169.59	164.33	165.07	149.87	149.49	152.36	150.95
C9	−5.06	23.74	21.63	16.36	7.55	2.19	1.16	−3.58	−5.01
H21	28.71	28.94	27.81	26.11	27.87	28.83	28.28	28.81	28.53
H22	29.59	29.79	28.41	26.63	28.59	29.49	28.99	29.47	29.29
H23	29.18	29.50	28.35	26.57	28.32	29.27	28.78	29.25	29.07
H24	29.36	29.62	28.48	26.74	28.62	29.50	28.98	29.49	29.28
H25	24.41	25.08	24.79	22.55	24.30	25.06	25.03	24.93	24.71
H26	27.43	27.85	27.00	25.20	26.70	27.77	27.26	27.41	27.28
H27	29.49	29.68	28.41	25.69	28.24	28.60	28.80	29.41	29.34
H28	28.16	28.53	27.20	24.50	26.67	27.34	27.56	28.13	28.09
H29	27.17	27.48	27.64	21.95	26.20	25.00	26.98	26.94	27.57
H30	23.71	24.29	24.20	21.73	23.31	24.12	24.15	24.21	24.14
H31	26.80	27.07	26.41	24.54	25.87	27.09	24.15	27.10	26.97
H32	27.86	28.07	27.39	25.49	26.99	28.14	28.03	28.15	28.02
H33	19.41	20.29	21.06	17.63	19.04	19.50	21.08	18.83	20.19
H34	28.00	28.31	27.38	25.73	27.34	28.43	27.97	28.04	27.96
H35	17.82	18.68	19.78	16.34	17.55	18.22	19.73	17.55	18.84
H36	29.27	29.55	28.61	26.05	28.63	28.86	29.16	29.26	29.15
H37	30.35	30.55	29.47	26.87	29.58	29.80	30.18	30.22	30.17
N11	127.49	150.00	145.66	139.06	136.39	132.64	130.44	131.25	129.95
N20	216.22	230.71	222.98	216.67	219.38	211.36	209.04	213.88	211.39
N5	124.24	148.45	143.94	137.47	134.97	131.12	128.70	131.12	128.65
O10	−77.85	−30.51	−32.96	−44.03	−57.32	−64.22	−65.74	−76.34	−78.18
O14	−227.46	−171.38	−169.76	−180.50	−200.41	−200.29	−201.76	−213.40	−214.86
O15	88.15	117.49	111.04	101.03	95.82	80.98	78.80	90.57	88.32
O18	−126.13	−75.69	−76.07	−86.73	−101.35	−106.33	−107.97	−119.42	−121.04
O19	65.20	97.85	92.87	82.91	76.74	63.17	60.69	72.76	70.19
O4	−73.30	−26.48	−28.69	−39.11	−51.73	−59.23	−61.58	−71.33	−74.00
S8	615.73	668.21	614.44	583.24	585.02	613.31	609.92	625.46	620.65

See footnote of Table S1.

Table S32: Ophthalmic acid, Cartesian coordinates.

Atm	x	y	z
C1	4.8383653	4.1728474	−4.7574939
C2	4.9697495	2.5503871	−2.3857879
C3	3.0212517	0.4138346	−2.4042563
H4	3.1619036	−0.6771205	−4.1603379
N5	0.4466896	1.3788983	−2.1769863
C6	−1.6219665	−0.1279535	−2.5778016
O7	−1.4390859	−2.3937383	−3.0684399
C8	−4.1329568	1.1434398	−2.0954978
C9	−5.1477367	0.5352874	0.5506668
C10	−3.3410156	1.1400921	2.7421268
H11	−1.5789494	0.0701970	2.6089207
N12	−4.5573192	0.4569724	5.1974583
C13	−2.6967015	3.9730902	2.8028319
O14	−3.8707919	5.1642230	4.7446901
O15	−1.3575718	5.0488106	1.2553076
C16	3.4397440	−1.3568641	−0.1229738
O17	3.7911919	−0.5900448	2.0390929
N18	3.0490512	−3.8873927	−0.6563374
C19	3.0773086	−5.7795786	1.3384314
C20	0.4974384	−6.1411750	2.5832926
O21	−1.4599010	−5.0840072	1.3166653
O22	0.1637675	−7.3888479	4.5015304
H23	6.3013992	5.6341167	−4.7022104
H24	3.0053772	5.1158418	−4.9251132
H25	5.1402626	3.0291619	−6.4545259
H26	4.7053921	3.7618274	−0.7237991
H27	6.8742737	1.7456813	−2.2357695
H28	0.1970667	3.1499542	−1.4398422
H29	−5.4689754	0.4638910	−3.5257148
H30	−3.9714712	3.1865417	−2.3790584
H31	−5.6253067	−1.4829953	0.6156486
H32	−6.9280763	1.5711929	0.8014254
H33	−5.2818097	−1.3348409	5.0125432
H34	−3.1308952	0.2019577	6.4938370
H35	−4.8244812	3.8293321	5.6512026
H36	2.5997426	−4.4089935	−2.4493627
H37	4.4393520	−5.2276414	2.7923381
H38	3.6410779	−7.5705491	0.4759579
H39	−0.9253932	−4.2858359	−0.2826585

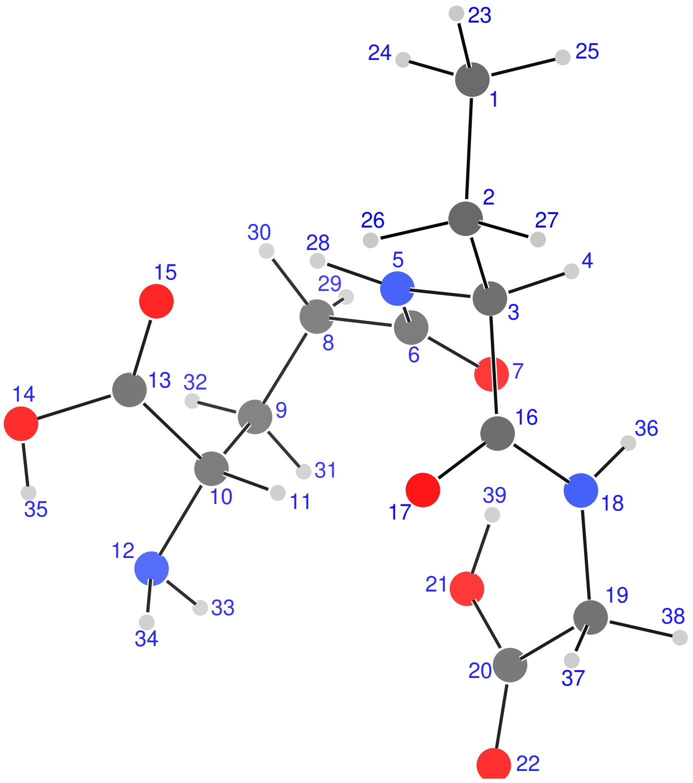

Table S33: Ophthalmic acid, nuclear magnetic shielding constants.

Atm	λ	GIAO	CSGT	DZ2	PZ2	GRRO $\bar{\alpha}$	GPRO $\bar{\beta}$	GRRO $\bar{\alpha}_{\mathrm{CE}}$	GPRO $\bar{\beta}_{\mathrm{CE}}$
C1	170.91	183.55	183.72	179.80	181.37	165.83	164.18	171.27	169.69
C10	127.30	143.35	145.08	141.37	139.25	126.73	125.45	128.46	127.06
C13	−10.20	19.35	15.66	10.71	1.67	−3.31	−4.15	−8.44	−9.63
C16	−5.95	−43.66	21.49	16.65	8.01	2.60	1.14	−3.17	−5.06
C19	137.64	152.12	151.03	146.94	145.45	132.49	131.10	136.11	134.02
C2	158.53	172.27	173.02	169.37	169.46	155.06	153.26	158.48	157.14
C20	−4.64	24.38	20.08	14.64	5.90	0.47	0.16	−4.65	−5.35
C3	122.51	138.18	138.79	134.98	132.66	120.62	118.73	122.34	120.40
C6	−10.64	19.05	17.03	12.35	3.28	−1.61	−3.38	−7.37	−9.52
C8	149.16	163.64	163.76	160.13	159.29	145.57	143.73	148.97	147.52
C9	151.43	165.69	167.24	163.62	162.96	149.15	147.47	152.55	151.26
H11	28.02	28.41	26.98	25.64	27.01	28.26	27.78	27.89	27.77
H23	30.29	30.52	28.82	26.63	29.11	29.74	29.37	30.26	30.01
H24	30.30	30.50	28.81	26.67	29.13	29.74	29.40	30.25	30.01
H25	30.83	31.02	29.30	27.10	29.63	30.19	29.87	30.72	30.51
H26	29.36	29.50	27.97	26.25	28.17	29.06	28.55	29.05	28.88
H27	29.98	30.22	28.79	26.99	28.97	29.87	29.39	29.86	29.74
H28	21.89	22.54	22.60	20.33	21.73	22.70	22.70	29.86	22.69
H29	29.45	29.74	28.68	27.05	28.74	29.74	29.25	29.72	29.54
H30	29.00	29.34	28.31	26.69	28.32	29.35	28.83	29.33	29.09
H31	29.44	29.68	28.37	26.69	28.46	29.42	28.94	29.40	29.22
H32	29.97	30.19	28.83	27.14	28.96	29.91	29.54	29.89	29.83
H33	29.77	30.10	28.84	26.47	29.07	29.30	29.67	29.70	29.64
H34	29.78	30.15	28.83	26.40	29.09	29.33	29.75	29.74	29.71
H35	19.03	30.15	20.27	17.53	18.86	19.65	20.63	18.95	19.76
H36	24.71	25.34	24.88	22.80	24.26	25.27	25.04	25.11	24.70
H37	26.89	27.28	26.67	24.88	26.29	27.40	27.16	27.41	27.13
H38	28.20	28.55	27.82	25.99	27.52	28.60	28.37	28.61	28.36
H39	20.50	21.33	21.75	18.93	20.19	20.94	21.66	20.26	20.94
H4	27.89	28.24	27.22	25.74	27.16	28.35	27.61	27.97	27.65
N12	207.34	222.86	218.44	212.38	214.69	207.03	204.68	209.54	206.98
N18	117.65	142.36	138.64	132.38	129.25	125.94	123.50	125.92	123.41
N5	100.68	126.03	122.39	116.24	112.21	110.03	107.17	108.64	106.72
O14	106.19	135.66	126.89	116.89	112.75	97.00	94.93	106.61	104.45
O15	−90.99	−43.66	−47.30	−57.83	−71.48	−78.07	−79.78	−91.11	−92.72
O17	−70.41	−24.48	−25.81	−36.38	−48.85	−56.44	−58.40	−68.56	−70.88
O21	108.38	138.00	132.18	122.17	118.09	102.32	99.86	111.92	109.42
O22	−116.64	−67.93	−70.94	−82.47	−97.19	−102.81	−102.99	−115.89	−116.07
O7	−51.46	−6.75	−7.82	−18.01	−29.85	−38.27	−40.92	−50.37	−53.34

See footnote of Table S1.

Table S34: Thyrotropin-releasing hormon, Cartesian coordinates.

Atm	x	y	z
C1	5.1686164	−6.7932838	2.3078644
C2	2.3943396	−7.2649020	1.6853130
C3	1.5068863	−5.2165011	0.4452200
N4	3.0647783	−3.2287640	0.9145550
C5	5.3036456	−3.9541486	2.3444499
C6	2.6209429	−0.6634983	0.4847845
O7	4.2294247	0.9583398	0.9984184
C8	0.0856034	0.1336727	−0.6766068
C9	0.4597599	0.6869540	−3.5007681
C10	−1.9979615	0.9172275	−4.8600362
C11	−3.2681925	2.9519338	−5.8706180
N12	−5.5199868	2.2066052	−6.9105437
C13	−5.6314990	−0.2470774	−6.5568477
N14	−3.5379902	−1.1230500	−5.3323238
N15	−0.7879069	2.3789960	0.6641737
C16	−2.2692944	2.2197191	2.7448568
O17	−3.1438468	0.3259210	3.7126795
C18	−2.8727355	4.6880937	4.0098467
C19	−0.8977930	5.9109597	5.0586067
C20	−0.7914185	8.6250674	3.8741088
C21	−3.0400297	8.6495382	2.1659806
O22	−3.7699545	10.3618939	0.8174338
N23	−4.0768879	6.2676535	2.4183224
C24	1.0961560	−5.6552508	−2.3354779
O25	−1.0285975	−5.2997131	−3.1509050
N26	3.1152397	−6.3465832	−3.7233627
H27	6.3488290	−7.5808791	0.8035755
H28	5.7167931	−7.6555802	4.1023266
H29	1.3768435	−7.4977958	3.4975092
H30	2.1418339	−9.0746389	0.6859514
H31	−0.4131004	−4.7775950	1.1410375
H32	5.1136717	−3.1948741	4.2592276
H33	7.0204920	−3.2019068	1.4747253
H34	−1.3363491	−1.3298685	−0.3881383
H35	1.5839863	−0.8024806	−4.3999967
H36	1.5450124	2.4386136	−3.7471812
H37	−2.6915794	4.9150851	−5.8983844
H38	−7.1592870	−1.4764910	−7.1335925
H39	−3.1978704	−2.9442147	−4.8201559
H40	−0.0368354	4.0876649	0.2629248
H41	−4.2630127	4.1130381	5.4599723
H42	0.9379370	4.9526929	4.8308379
H43	−1.1657852	6.1188534	7.1163033
H44	0.9421442	8.8589285	2.7787985
H45	−0.9688070	10.0679552	5.3380877
H46	−5.6234569	5.7678812	1.3962961
H47	4.8119690	−6.6006641	−2.8992906
H48	2.9052731	−6.6735272	−5.5899584

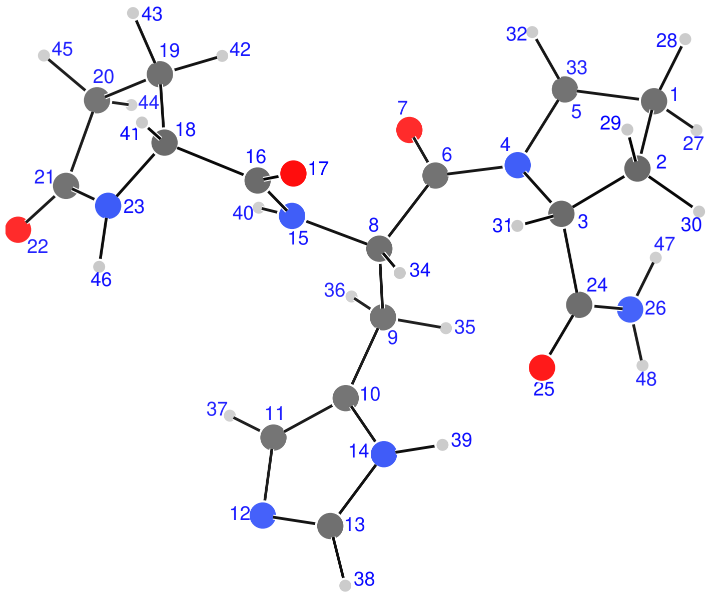

Table S35: Thyrtropin-releasing hormone, nuclear magnetic shielding constants.

Atm	λ	GIAO	CSGT	DZ2	PZ2	GRRO $\bar{\alpha}$	GPRO $\bar{\beta}$	GRRO $\bar{\alpha}_{\mathrm{CE}}$	GPRO $\bar{\beta}_{\mathrm{CE}}$
C1	158.70	172.34	172.70	169.11	168.88	154.66	152.57	158.06	156.39
C10	49.17	73.21	69.92	65.02	58.84	50.17	48.83	47.77	46.78
C11	43.85	68.95	64.85	59.71	53.47	44.98	43.82	46.14	44.84
C13	36.44	62.33	58.11	52.85	46.31	38.47	37.25	37.27	35.79
C16	−5.93	23.14	19.59	14.82	5.70	0.83	−0.98	−4.95	−7.19
C18	132.73	148.62	146.93	142.95	140.72	128.85	126.30	130.54	127.88
C19	167.17	180.01	180.55	176.88	176.76	162.51	160.34	165.89	164.09
C2	160.68	174.28	173.42	169.86	169.49	155.64	153.01	159.02	156.76
C20	154.59	168.29	169.00	165.12	164.57	150.43	148.98	153.83	152.76
C21	−3.80	24.98	21.93	16.61	7.70	2.32	1.68	−3.48	−4.59
C24	−7.73	21.43	18.59	13.83	4.98	−0.09	−1.94	−5.87	−8.22
C3	131.50	147.39	144.81	140.99	138.83	127.03	124.05	128.71	125.64
C5	139.02	154.06	153.04	149.30	147.90	135.26	132.95	138.87	135.93
C6	−13.14	16.43	14.33	9.67	0.61	−4.30	−6.22	−10.09	−12.43
C8	129.00	143.71	143.73	139.98	137.73	125.71	123.43	127.41	125.01
C9	154.13	167.53	166.64	163.06	162.28	148.82	146.41	152.20	150.08
H27	29.72	30.00	28.68	26.86	28.61	29.73	28.93	29.71	29.25
H28	29.37	29.63	28.37	26.63	28.39	29.48	28.74	29.45	29.05
H29	28.85	29.06	27.89	26.31	27.91	29.06	28.30	29.04	28.58
H30	29.31	29.62	28.33	26.82	28.40	29.55	28.76	29.52	29.03
H31	26.45	26.79	26.23	24.86	26.05	27.42	26.53	27.04	26.52
H32	28.32	28.47	27.53	25.88	27.45	28.65	27.83	28.66	27.84
H33	27.71	27.95	26.95	25.34	26.74	28.07	27.25	28.07	27.26
H34	26.55	26.93	26.16	24.78	26.02	27.26	26.37	26.89	26.33
H35	29.01	29.33	28.06	26.64	28.10	29.31	28.40	29.28	28.58
H36	28.89	29.19	28.08	26.62	28.11	29.20	28.37	29.17	28.56
H37	24.41	24.61	24.53	22.31	23.77	25.02	24.81	24.56	24.40
H38	23.90	24.11	24.13	21.90	23.40	24.60	24.46	23.98	23.82
H39	20.23	20.88	20.77	18.35	19.47	20.75	20.75	20.56	20.30
H40	26.14	26.62	25.92	24.07	25.56	26.64	26.28	26.72	26.22
H41	27.12	27.36	26.89	25.35	26.48	28.01	27.45	27.60	27.43
H42	29.33	29.65	28.62	27.03	28.55	29.71	29.17	29.68	29.42
H43	28.85	29.12	28.19	26.47	28.02	29.25	28.77	29.22	29.05
H44	29.10	29.37	28.47	26.56	28.14	29.28	29.04	29.25	29.31
H45	29.19	29.46	28.56	26.66	28.34	29.40	29.06	29.37	29.33
H46	27.00	27.39	26.58	24.38	26.09	27.03	27.28	26.86	26.78
H47	26.34	26.75	26.01	23.63	25.64	26.38	26.41	26.20	25.87
H48	26.62	26.99	26.22	23.79	25.75	26.53	26.73	26.35	26.13
N12	−52.83	−10.28	−14.66	−21.83	−33.09	−28.29	−30.36	−55.87	−56.41
N14	70.47	101.12	95.93	89.08	83.80	82.74	80.29	74.83	72.75
N15	136.23	158.42	153.15	147.15	144.59	140.91	137.84	139.51	137.32
N23	120.32	144.51	138.02	131.56	128.24	124.93	122.52	124.90	122.37
N26	162.12	182.95	176.16	169.80	169.55	163.90	161.40	160.86	157.67
N4	115.44	138.23	133.41	127.20	123.49	120.48	117.49	120.46	117.47
O17	−86.54	−39.54	−42.64	−53.18	−66.60	−73.35	−75.37	−85.47	−87.86
O22	−72.23	−25.39	−25.22	−36.70	−48.84	−57.05	−57.44	−69.19	−70.03
O25	−78.86	−31.43	−32.66	−43.07	−56.04	−63.26	−65.74	−75.38	−78.25
O7	−119.03	−67.63	−70.90	−81.27	−96.45	−101.39	−103.76	−113.52	−116.26

See footnote of Table S1.

Table S36: Nuclear magnetic shielding constants calculated using $\bar{\alpha}$ and $\bar{\beta}$ for a selection of molecules common to the Schattenberg-Kaupp NS372 benchmark set (*J. Chem. Theory Comput.* **2021**, *17*, 7602-7621) and the reference benchmark by Jensen (*J. Chem. Theory Comput.* **2018**, *14*, 4651-4661). Absolute deviations with respect to accurate forecasts (λ) are reported in the last three columns.

Mol	Atm	λ	GIAO	GRRO	GPRO	$\lvert\lambda - \text{GIAO}\rvert$	$\lvert\lambda - \text{GRRO}\rvert$	$\lvert\lambda - \text{GPRO}\rvert$
C_2H_4	C1	49.59	76.03	54.29	56.63	26.44	4.70	7.03
	H3	25.93	26.08	25.30	27.94	0.16	0.63	2.02
C_2H_4O	O1	355.66	362.88	297.05	298.50	7.22	58.61	57.16
	C2	144.39	159.42	142.14	142.27	15.03	2.25	2.12
	H4	29.36	29.52	28.58	30.34	0.16	0.78	0.98
C_3H_4	C1	183.79	195.34	179.49	181.24	11.55	4.30	2.55
	C2	62.92	87.46	60.91	62.74	24.54	2.01	0.17
	H4	24.06	24.34	23.43	25.21	0.27	0.63	1.14
	H6	30.71	30.88	29.87	31.79	0.17	0.84	1.08
CH_4	C1	191.12	201.78	182.04	184.43	10.66	9.07	6.69
	H2	31.47	31.65	29.59	32.93	0.18	1.88	1.46
CO	C1	−25.46	10.76	−14.77	−13.54	36.22	10.69	11.92
	O2	−88.36	−38.42	−79.16	−77.67	49.95	9.20	10.70
H_2CCO	C1	185.43	193.73	164.12	167.95	8.30	21.31	17.48
	C2	−23.20	11.20	−21.41	−16.08	34.40	1.79	7.12
	O3	−32.92	−0.21	−43.01	−38.54	32.70	10.09	5.62
	H4	29.25	29.63	28.07	31.25	0.39	1.18	2.01
H_2CO	C1	−21.41	10.80	−13.07	−9.12	32.21	8.34	12.29
	O2	−462.10	−400.18	−428.29	−422.86	61.92	33.81	39.24
	H3	21.80	22.20	21.83	25.32	0.41	0.04	3.52
H_2O	O1	328.23	337.75	253.21	260.29	9.52	75.02	67.93
	H2	30.76	31.56	28.51	37.22	0.80	2.25	6.46
HCN	C1	67.26	94.48	63.33	67.94	27.22	3.93	0.68
	N2	−54.15	−9.20	−21.30	−18.85	44.95	32.84	35.30
	H3	29.12	28.96	28.10	31.91	0.16	1.02	2.79
N_2	N1	−103.81	−54.87	−76.90	−75.41	48.94	26.91	28.40
N_2O	N1	71.86	91.79	77.26	79.69	19.92	5.40	7.83
	N2	−22.97	9.41	−7.25	−2.91	32.37	15.72	20.06
	O3	173.25	179.68	147.05	150.68	6.42	26.20	22.57
NH_3	N1	261.31	271.76	244.79	249.41	10.45	16.53	11.90
	H2	31.60	31.92	29.10	35.71	0.32	2.51	4.11

See footnote of Table S1.

Table S37: Sum of squares of the residuals obtained from isotropic shielding constants computed by various methods at the BHandHLYP/6-31+G(d,p) compared with the limit values λ. The degrees of freedom df are also reported.

		GIAO	GRRO	GPRO	GRROce	GPROce
C	SSR	49716.47	1350.07	1591.92	145.76	95.15
	df	107	106	106	82	82
H	SSR	32.95	69.86	145.65	15.24	46.63
	df	197	196	196	168	168
N	SSR	11250.10	1066.07	1027.22	113.32	74
	df	28	27	27	17	17
O	SSR	74141.00	8837.46	9450.23	42.98	56.03
	df	47	46	46	47	39

Table S38: Values of the ratio F=(SSR_1/df_1)/(SSR_2/df_2) computed from the data of Table 1 for comparison of the pairs of models indicated in the first line. The critical value F_{crit} corresponds to the value of F exceeded with a probability of 0.01 under the hypothesis that the residuals of the two models follow the same distribution.

		$\frac{GIAO}{GRRO}$	$\frac{GIAO}{GPRO}$	$\frac{GIAO}{GRROce}$	$\frac{GIAO}{GPROce}$	$\frac{GRRO}{GRROce}$	$\frac{GPRO}{GPROce}$
C	F	36.48	30.94	261.40	400.45	7.17	12.94
	F_{crit}	1.57	1.57	1.64	1.64	1.64	1.64
H	F	0.47	0.23	1.84	0.60	3.93	2.68
	F_{crit}	1.40	1.40	1.42	1.42	1.42	1.42
N	F	10.19	10.57	61.62	94.32	6.05	8.92
	F_{crit}	2.45	2.45	2.93	2.93	2.94	2.94
O	F	8.22	7.69	1443.38	1107.29	175.63	144.08
	F_{crit}	1.97	1.97	2.05	2.05	2.05	2.05

Table S39: BHandHLYP/6-31+G(d,p) mean absolute deviation in ppm for ^{13}C chemical shift with reference to propane.

AA	GIAO	GRRO $\bar{\alpha}$	GPRO $\bar{\beta}$	GRRO $\bar{\alpha}_{CE}$	GPRO $\bar{\beta}_{CE}$
Gly	9.53	5.77	6.18	0.49	1.32
Ala	7.05	5.47	4.67	0.66	0.91
Val	4.81	4.82	3.89	0.85	1.52
Leu	4.37	4.97	3.44	1.26	1.40
Ile	4.25	4.56	3.78	0.85	1.53
Pro	5.31	4.29	2.57	0.35	2.58
Ser	7.84	5.95	5.43	0.40	1.76
Thr	6.30	5.54	4.37	0.68	1.44
Asn	9.68	7.03	5.86	0.37	2.04
Gln	8.00	6.62	5.36	0.82	1.35
Cys	7.62	6.24	5.97	0.48	0.93
Met	5.25	5.18	4.36	1.30	0.64
Phe	11.24	8.19	5.92	1.27	1.36
Tyr	11.03	7.45	5.55	1.20	1.80
Trp	10.51	6.51	4.64	0.83	2.62
Asp	9.56	6.80	5.85	0.35	2.00
Glu	7.81	5.93	5.01	0.57	1.92
His	9.84	6.13	4.94	0.54	1.90
Lys	4.86	5.24	3.52	1.47	0.89
Arg	6.94	5.83	4.16	0.99	1.48
min	4.25	4.29	2.57	0.35	0.64
max	11.24	8.19	6.18	1.47	2.62
median	7.72	5.88	4.80	0.75	1.50

Table S40: BHandHLYP/6-31+G(d,p) mean absolute deviation for ^{1}H chemical shift with reference to propane.

AA	GIAO	GRRO $\bar{\alpha}$	GPRO $\bar{\beta}$	GRRO $\bar{\alpha}_{\mathrm{CE}}$	GPRO $\bar{\beta}_{\mathrm{CE}}$
Gly	0.28	1.10	1.15	0.53	0.47
Ala	0.20	0.99	0.57	0.57	1.01
Val	0.14	0.99	0.93	0.67	1.51
Leu	0.13	0.96	1.14	0.60	1.75
Ile	0.12	0.99	1.00	0.62	1.61
Pro	0.15	1.44	0.58	0.71	1.41
Ser	0.24	1.15	0.63	0.53	1.02
Thr	0.19	1.10	0.61	0.62	1.26
Asn	0.28	1.41	0.50	0.71	1.22
Gln	0.23	1.34	0.46	0.66	1.22
Cys	0.23	0.90	0.58	0.36	1.01
Met	0.22	0.79	0.79	0.58	1.25
Phe	0.17	1.64	0.75	0.80	1.71
Tyr	0.20	1.53	0.67	0.73	1.51
Trp	0.18	1.59	0.66	0.74	1.75
Asp	0.26	1.36	0.54	0.69	1.17
Glu	0.21	1.39	0.53	0.71	1.18
His	0.21	1.54	0.64	0.74	1.34
Lys	0.13	0.97	0.78	0.40	1.69
Arg	0.23	1.18	0.53	0.60	1.46
min	0.12	0.79	0.46	0.36	0.47
max	0.28	1.64	1.15	0.80	1.75
median	0.21	1.17	0.64	0.64	1.30

Table S41: BHandHLYP/6-31+G(d,p) ^{13}C MAD in ppm for nine additional conformations of Alanine obtained rotating independently the $-CH_3$, $-COOH$, and $-NH_2$ groups about the single bonds connecting the three fragments to the α carbon (dihedral angle ϕ in degrees).

Group	ϕ	GIAO	CSGT	GRRO $\bar{\alpha}$	GPRO $\bar{\beta}$	GRRO $\bar{\alpha}_{CE}$	GPRO $\bar{\beta}_{CE}$
CH_3	0	19.80	19.14	3.42	3.89	0.73	0.62
	40	19.54	18.76	3.63	4.15	0.51	0.28
	80	19.72	19.03	3.46	3.95	0.65	0.48
	120	19.81	19.15	3.42	3.89	0.74	0.63
COOH	40	19.86	19.29	3.52	3.93	0.89	0.90
	80	19.87	19.52	3.27	3.71	1.14	1.02
	120	20.08	19.59	3.06	3.50	1.21	1.07
NH_2	40	19.50	18.73	3.74	4.22	0.37	0.21
	80	19.48	18.77	3.77	4.18	0.34	0.38
	120	19.39	18.74	3.62	4.06	0.49	0.30
	min	19.39	18.73	3.06	3.50	0.34	0.21
	max	20.08	19.59	3.77	4.22	1.21	1.07
	median	19.76	19.08	3.49	3.94	0.69	0.55

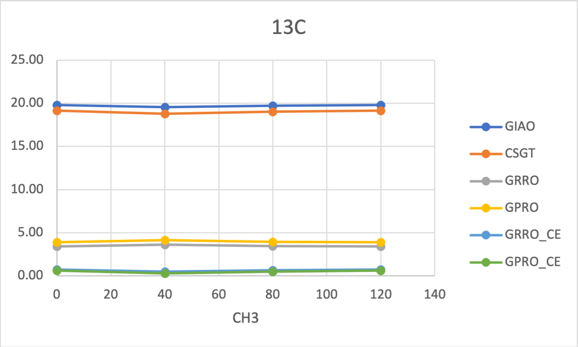

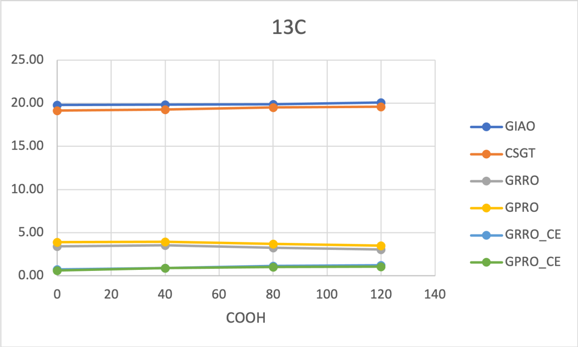

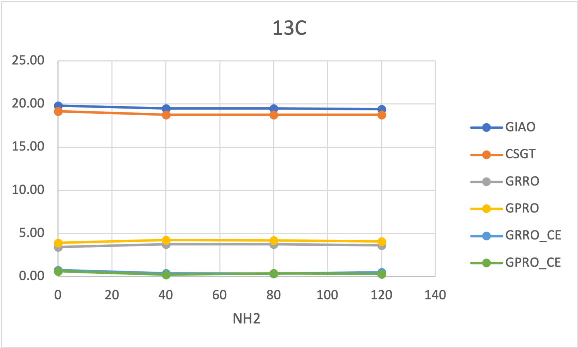

Table S42: BHandHLYP/6-31+G(d,p) ^{1}H MAD in ppm for nine additional conformations of Alanine obtained rotating independently the $-CH_3$, $-COOH$, and $-NH_2$ groups about the single bonds connecting the three fragments to the α carbon (dihedral angle ϕ in degrees).

Group	ϕ	GIAO	CSGT	GRRO $\bar{\alpha}$	GPRO $\bar{\beta}$	GRRO $\bar{\alpha}_{CE}$	GPRO $\bar{\beta}_{CE}$
	0	0.39	1.21	0.56	0.58	0.22	0.58
	40	0.40	1.08	0.50	0.61	0.14	0.64
CH_3	80	0.41	1.06	0.49	0.62	0.16	0.69
	120	0.39	1.22	0.57	0.59	0.22	0.58
	40	0.33	1.10	0.57	0.51	0.39	0.56
COOH	80	0.34	1.11	0.58	0.53	0.39	0.44
	120	0.35	1.10	0.57	0.60	0.35	0.47
	40	0.37	1.15	0.51	0.60	0.14	0.63
NH_2	80	0.39	1.19	0.56	0.66	0.25	0.72
	120	0.39	1.10	0.46	0.67	0.23	0.68
	min	0.33	1.06	0.46	0.51	0.14	0.44
	max	0.41	1.22	0.58	0.67	0.39	0.72
	median	0.39	1.11	0.56	0.60	0.23	0.60

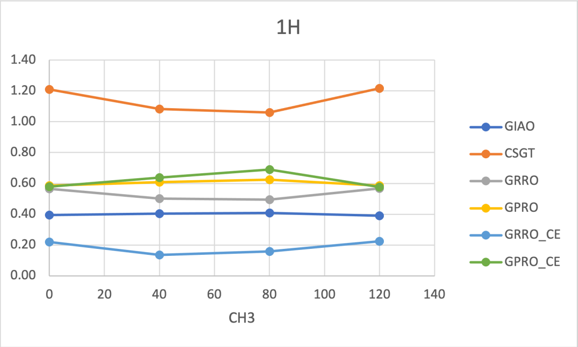

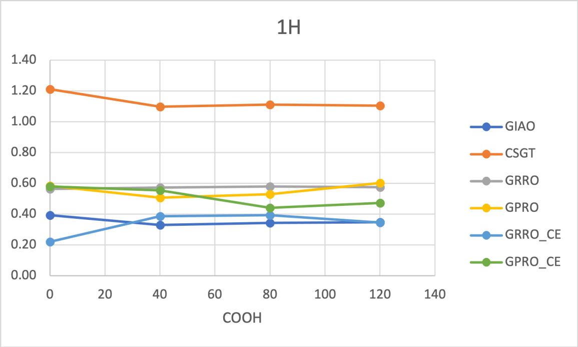

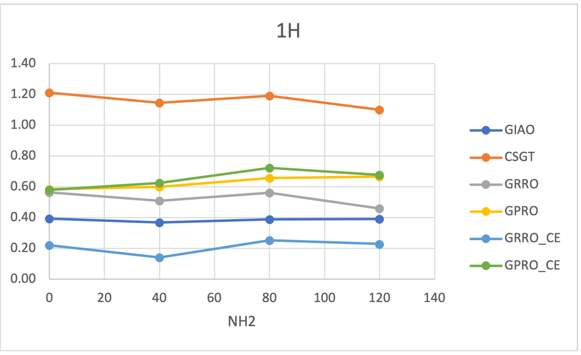

Table S43: BHandHLYP/6-31+G(d,p) ^{15}N MAD in ppm for nine additional conformations of Alanine obtained rotating independently the $-CH_3$, $-COOH$, and $-NH_2$ groups about the single bonds connecting the three fragments to the α carbon (dihedral angle ϕ in degrees).

Group	ϕ	GIAO	CSGT	GRRO $\bar{\alpha}$	GPRO $\bar{\beta}$	GRRO $\bar{\alpha}_{CE}$	GPRO $\bar{\beta}_{CE}$
	0	17.25	12.66	0.75	0.54	3.28	1.78
	40	18.04	13.71	1.74	0.51	4.27	2.82
CH_3	80	17.94	13.22	1.28	0.05	3.81	2.36
	120	17.24	12.65	0.74	0.56	3.26	1.76
	40	16.78	9.99	2.22	3.10	0.32	0.75
COOH	80	16.61	8.65	3.56	4.56	1.02	2.17
	120	16.65	8.38	3.71	4.75	1.17	2.36
	40	16.36	11.91	0.01	1.42	2.51	0.91
NH_2	80	16.01	9.15	2.81	3.89	0.28	1.58
	120	17.28	8.74	3.16	4.51	0.63	2.16
	min	16.01	8.38	0.01	0.05	0.28	0.75
	max	18.04	13.71	3.71	4.75	4.27	2.82
	median	17.01	10.95	1.98	2.26	1.84	1.97

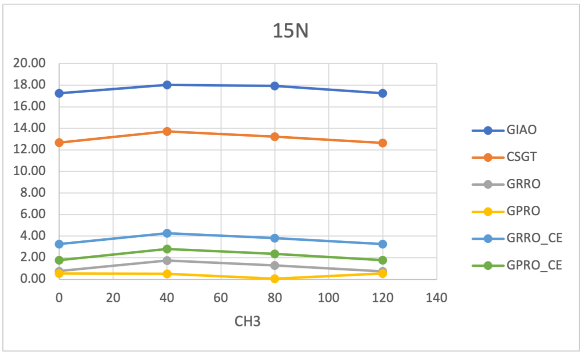

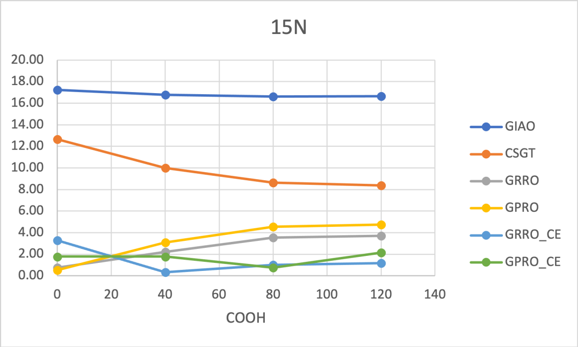

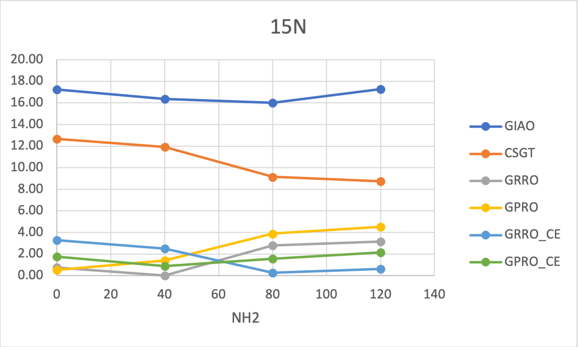

Table S44: BHandHLYP/6-31+G(d,p) ^{17}O MAD in ppm for nine additional conformations of Alanine obtained rotating independently the $-CH_3$, $-COOH$, and $-NH_2$ groups about the single bonds connecting the three fragments to the α carbon (dihedral angle ϕ in degrees).

Group	ϕ	GIAO	CSGT	GRRO $\bar{\alpha}$	GPRO $\bar{\beta}$	GRRO $\bar{\alpha}_{CE}$	GPRO $\bar{\beta}_{CE}$
	0	38.66	33.09	11.21	11.91	0.14	0.65
	40	38.19	33.11	11.22	11.87	0.18	0.61
CH_3	80	38.81	33.52	11.83	12.48	0.60	1.22
	120	38.66	33.12	11.23	11.93	0.17	0.67
	40	38.09	32.51	14.64	15.25	3.30	4.02
COOH	80	38.21	31.97	14.13	14.68	2.79	3.45
	120	38.16	31.36	13.81	14.33	2.47	3.10
	40	38.09	33.33	11.33	12.02	0.39	0.76
NH_2	80	38.67	33.08	11.62	12.26	0.28	1.01
	120	37.89	32.03	12.30	13.00	0.96	1.73
	min	37.89	31.36	11.21	11.87	0.14	0.61
	max	38.81	33.52	14.64	15.25	3.30	4.02
	median	38.20	33.09	11.73	12.37	0.49	1.11

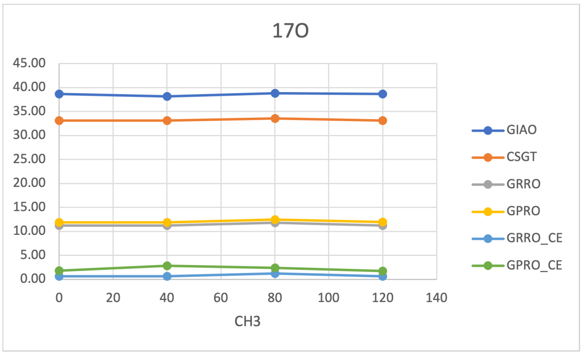

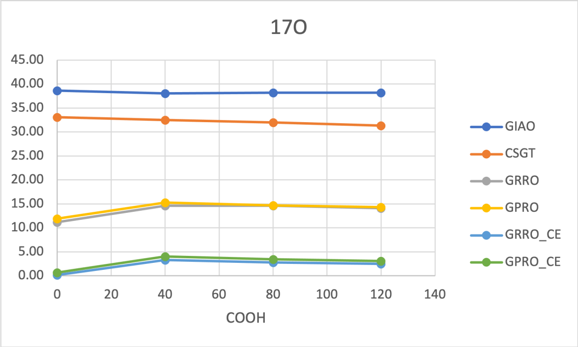

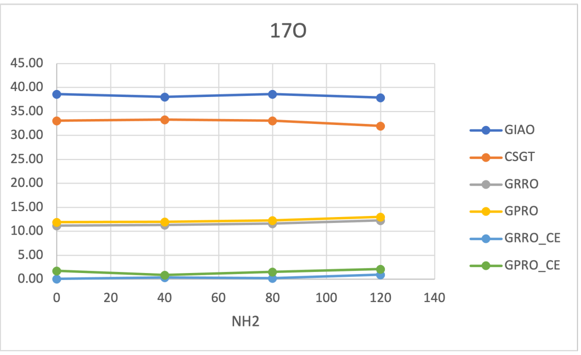

Supplement: Supplementary file 1 — jp2c08271_si_001.pdf [file jp2c08271_si_001.pdf]
